# Effects of Interprofessional Collaborative Practice in Older Adults’ Hip Fractures Care: A Systematic Review and Meta‐Analysis of Randomized Controlled Trials

**DOI:** 10.1002/nur.70125

**Published:** 2026-09-08

**Authors:** Santo Imanuel Tonapa, I‐Chin Huang, Bih‐O Lee, Wendy Chaboyer

**Affiliations:** ^1^ School of Nursing, Galuh University Ciamis Indonesia; ^2^ College of Nursing, Kaohsiung Medical University Kaohsiung Taiwan; ^3^ Biomedical Artificial Intelligence Academy, Kaohsiung Medical University Kaohsiung Taiwan; ^4^ Graduate Institute of Medicine, Kaohsiung Medical University Kaohsiung Taiwan; ^5^ Center for Medical Education and Humanizing Health Professional Education Kaohsiung Medical University Kaohsiung Taiwan; ^6^ Department of Nursing Chung Hwa University of Medical Technology Tainan Taiwan; ^7^ School of Nursing and Midwifery, Griffith University Brisbane Queensland Australia; ^8^ NHMRC Centre of Research Excellence in Wiser Wound Care Griffith University Queensland Queensland Australia

**Keywords:** activities of daily living, aged, hip fractures, interprofessional collaboration, interprofessional relations, meta‐analysis

## Abstract

This study aimed to evaluate the effectiveness of interprofessional collaborative practice interventions in improving activities of daily living, quality of life, length of stay, and in reducing mortality among older adults with hip fractures. Systematic review and meta‐analysis of randomized controlled trials. Eight databases including AgeLine, PubMed, Embase, CINAHL Ultimate, Scopus, MEDLINE Ultimate, Web of Science, and the Cochrane Library were searched from inception to November 2025. Two reviewers independently screened studies and assessed risk of bias using Cochrane Risk‐of‐Bias 2.0. Random‐effects meta‐analyses were performed. Certainty of evidence was evaluated using GRADE. Twelve randomized controlled trials met inclusion criteria. Interventions typically involved nurses working alongside geriatricians, orthopedic surgeons, and physiotherapists within structured co‐management pathways. Interprofessional collaborative practice was associated with a shorter hospital stay by a mean of 2.26 days. Activities of daily living improved by 4.86 points at 6 months and 3.92 points at 12 months on the Barthel Index. Health‐related quality of life improved modestly (Hedges’ G = 0.194), though this was not robust to sensitivity analysis. Mortality evidence was inconclusive at in‐hospital and 6‐month follow‐up. Certainty of evidence ranged from very low to low across outcomes. Interprofessional collaborative practice appears to improve functional recovery and may shorten hospital stay among older adults with hip fractures, though any mortality benefit remains unproven. Clinicians should consider adopting interprofessional hip fracture pathways spanning team rounds, early mobilization, medication reconciliation, and discharge planning, while future trials clarify effects on survival.

Trial Registration: PROSPERO registration number: CRD42024573834

## 1 Introduction

Hip fractures are a leading cause of disability, dependency, and premature mortality among older adults worldwide (Chiang et al. 2022), with global incidence projected to rise from 1.26 million cases in 1990 to 4.5 million by 2050 (Veronese and Maggi 2018) and the total annual number of fractures expected to nearly double by then due to population aging, despite more than 10‐fold geographic variation in risk (Feng et al. 2024; Kanis et al. 2012; Sing et al. 2023). Beyond their clinical impact, hip fractures impose a substantial economic burden, reflected in high rates of hospitalization, aged‐care transition, and readmission (Australian Institute of Health and Welfare 2018, 2023), a shift of costs toward primary and community care under standardized pathways (Haugan et al. 2021), and long‐term rehabilitation and caregiver burden (Goh et al. 2025). These trends highlight an urgent need for strategies to prevent fractures and optimize survival, recovery, and long‐term quality of life.

Older adults with hip fractures commonly present with multiple chronic conditions and frailty, making recovery more complex and prolonged. Post‐fracture complications, such as infections, delirium, venous thromboembolism, and delayed mobilization, are common and strongly associated with adverse outcomes. These complications contribute to excess length of stay and higher mortality. One‐year mortality following hip fracture ranges from 8.4% to 36% and remains high even in recent data, with disparities persisting by socioeconomic deprivation (Rivera‐Zarazúa et al. 2021; Webster et al. 2025). Reducing mortality in this vulnerable group requires not only surgical expertise but also coordinated and comprehensive management strategies before, during, and after hospitalization.

While survival is a key outcome, the broader impact of hip fractures on the daily lives of older adults must also be acknowledged. Most patients with hip fractures already experience some limitations in function, but surgery often leads to a further decline in mobility and independence (Pioli et al. 2018). Functional recovery is often incomplete, and many patients fail to return to their pre‐fracture level of performance. This decline is most clearly observed in basic and instrumental activities of daily living, such as walking, dressing, toileting, and managing household tasks (Abeygunasekara et al. 2021). Patients often describe recovery as regaining “normal life,” yet vulnerability, dependence, and caregiver burden can persist well beyond discharge (Beer et al. 2022).

In the standard care model, services for patients with hip fractures are often fragmented, with limited coordination between orthopedic, geriatric, nursing, and rehabilitation teams. Such models tend to focus on the surgical episode, overlooking the functional and psychosocial needs that drive long‐term outcomes; addressing these needs requires coordinated input from multiple health professionals across stages of care (Folbert et al. 2017; Drew et al. 2024). Clinical guidelines endorse interprofessional co‐management for this population, although real‐world implementation remains limited (Australian Institute of Health and Welfare 2018; Ma et al. 2019; Roberts et al. 2022). Systematic reviews confirm that bundled, multidisciplinary approaches achieve greater recovery gains than single‐component interventions (Phang et al. 2023). In this context, interprofessional collaborative practice has emerged as a promising approach for addressing the multifaceted needs of older adults with hip fractures.

Interprofessional collaborative practice involves professionals from diverse backgrounds, such as orthopedic surgeons, geriatricians, nurses, physiotherapists, and social workers, working together with patients and caregivers to plan and deliver care. The World Health Organization defines interprofessional collaboration as a process in which multiple health workers from different professional backgrounds work together with patients, families, and communities to deliver the highest quality of care (World Health Organization 2010). This model fosters shared decision‐making, continuous communication, and coordinated interventions tailored to individual patient needs. In the context of hip fracture care, interprofessional collaborative practice‐based interventions may involve early geriatric assessment, coordinated rehabilitation, pain and medication management, discharge planning, and follow‐up support in the community (Riemen and Hutchison 2016).

Clinical evidence supports the effectiveness of interprofessional collaborative practice in improving a broad range of outcomes for older adults with hip fractures. For instance, one hospital in the Netherlands observed a reduction in 1‐year mortality from 14% under usual care to just 4.7% after implementing a specialist hip fracture care model (Folbert et al. 2017). Central to these outcomes are early mobilization, individualized rehabilitation, and continuity of care—hallmarks of a structured, team‐based model that addresses the complex, multidimensional needs of this population.

Despite the increasing implementation of interprofessional care models in clinical practice, the extent to which these approaches improve measurable outcomes in older adults with hip fractures remains uncertain. While individual randomized controlled trials have suggested benefits of interprofessional collaborative practice (Swanson et al. 1998; Shyu et al. 2008; Wu et al. 2023), variation in intervention design, team composition, and outcome measurement has made it difficult to draw firm conclusions. Existing reviews address adjacent aspects of hip fracture care, including mobilization, enhanced recovery, treatment trends, and patient perspectives (Agarwal et al. 2024; Beer et al. 2022; Liu et al. 2021; Sarkies et al. 2023; Sing et al. 2023). Interprofessional collaborative practice in hip fracture care also overlaps conceptually with established orthogeriatric co‐management and comprehensive geriatric assessment models, which have been examined in prior reviews. However, the extent to which trials implement explicit collaborative focal areas and the implications of this variation for core outcomes remain insufficiently synthesized. A systematic synthesis of randomized controlled trial evidence is therefore needed to clarify the effectiveness of interprofessional collaborative practice for this population and to inform the design and implementation of future hip fracture care pathways.

This study aimed to systematically review and meta‐analyze the effects of interprofessional collaborative practice–based interventions among older adults with hip fractures. By synthesizing evidence from available trials data, this review seeks to inform best practices for multidisciplinary care delivery, with the ultimate goal of improving survival, functional independence, and quality of life for older patients recovering from hip fracture.

## 2 Methods

### 2.1 Study Design and Registration

This systematic review and meta‐analysis was conducted in accordance with the Preferred Reporting Items for Systematic Reviews and Meta‐Analyses (PRISMA) 2020 guidelines (Page et al. 2021). The protocol was registered in the International Prospective Register of Systematic Reviews (PROSPERO) under registration number CRD42024573834.

### 2.2 Eligibility Criteria

Randomized controlled trials were eligible if they enrolled adults aged 60 years or older with a hip fracture and evaluated an intervention consistent with the World Health Organization concept of interprofessional collaborative practice (World Health Organization 2010). Interprofessional collaborative practice was defined as a holistic, team‐based approach that coordinates medical and surgical management with rehabilitation, psychosocial support, complication prevention, discharge planning, and, when applicable, home or community follow‐up (Supporting Information S1: Table 1). Comparators were usual or conventional care (e.g., single‐specialty or orthopedic‐led care) or waitlist control (Supporting Information S1: Table 2).

Trials testing single‐modality interventions (e.g., physiotherapy only, nutritional supplementation alone, anesthetic protocols) were excluded because they did not reflect holistic interprofessional collaborative practice construct. Additional exclusions were non–peer reviewed reports, non‐English language publications, conference abstracts, theses, pilot or feasibility studies, and reviews. Trials evaluating enhanced recovery after surgery protocols were also excluded because, although multidisciplinary in personnel, such protocols are defined by adherence to a standardized perioperative pathway rather than a jointly negotiated interprofessional care plan, and do not meet the operational definition of interprofessional collaborative practice adopted in this review (Supporting Information S1: Table 3).

### 2.3 Information Sources and Search Strategy

A comprehensive search of AgeLine, PubMed, Embase, CINAHL ultimate, Scopus, Medline ultimate, Web of Science, and Cochrane library databases was conducted, covering all records from inception to November 2025. The search strategy combined both Medical Subject Headings (MeSH) and free‐text terms related to “hip fracture,” “older adults,” “interprofessional collaboration,” “randomized controlled trial.” Additionally, reference lists across included studies were explored. The complete search strategy for each database is provided in Supporting Information S1: Table 4.

### 2.4 Study Selection

All search results were imported into EndNote for de‐duplication and screening. Two reviewers independently screened titles and abstracts to identify potentially eligible studies, followed by a full‐text review to determine final inclusion. Any disagreements regarding study selection were resolved through discussion and consensus, with the involvement of a third reviewer when necessary.

### 2.5 Data Extraction

A standardized data extraction form was developed to collect relevant data from each study which was pilot tested prior to use. Two reviewers independently extracted information, including study characteristics (author, year, country, sample size), participant demographics (age), intervention details (duration, content, professionals involved), comparator characteristics, outcomes assessed, and time points of measurement. Statistical data such as means, standard deviations, and effect sizes were also extracted. Discrepancies in the extracted data were discussed and resolved through consensus.

Outcome reporting in the included trials was mapped against the Core Outcome Set (COS) recommended for hip fracture research, comprising mortality, pain, activities of daily living, mobility, and quality of life. Likewise, the focal areas of each intervention (e.g., comprehensive geriatric assessment, early surgery, mobilization, rehabilitation, nutrition, psychosocial support, discharge planning, home or community rehabilitation) were mapped to illustrate the multidimensional nature of interprofessional collaborative practice.

### 2.6 Risk of Bias Assessment

To assess the methodological quality of included studies, we used the Cochrane Risk of Bias 2.0 tool. Two reviewers independently evaluated each study across the five risks of bias domains: the randomization process, deviations from intended interventions, missing outcome data, measurement of the outcome, and selection of the reported result. Conflicts in risk of bias ratings were resolved through discussion and consensus (Flemyng et al. 2020).

### 2.7 Data Synthesis and Statistical Analysis

All statistical analyses were conducted using Comprehensive Meta‐Analysis software (version 4.0; Biostat Inc., Englewood, NJ, USA). A random‐effects model based on the DerSimonian and Laird method was applied to account for clinical and methodological heterogeneity among the included studies. The DerSimonian and Laird estimator was chosen for consistency with the software used; as a pre‐specified sensitivity analysis, all outcomes were re‐estimated using restricted maximum likelihood (REML) estimation with the Hartung–Knapp variance correction to assess robustness of conclusions. For continuous outcomes, mean differences (MDs) with 95% confidence intervals (CIs) were calculated. One trial (Prestmo et al. 2015) reported activities of daily living on the original 0–20 Barthel Index; scores were rescaled to the 0–100 metric by multiplication by five prior to pooling, consistent with the linear scaling property of the instrument. For dichotomous outcomes such as mortality, pooled risk ratios (RRs) with 95% CIs were computed using the reported event counts and total sample sizes for each group. Statistical heterogeneity was evaluated using the I^2^ statistic, with values exceeding 50% interpreted as indicating substantial heterogeneity (Borenstein 2024). One included trial (Panella et al. 2018) employed a cluster randomized design, randomizing at the hospital level across 26 hospitals. Effective sample sizes were calculated by dividing observed N by the design effect (DEFF = 1.342), derived from the reported intraclass correlation coefficient (ICC = 0.018) and mean cluster size (20 patients), prior to meta‐analysis (Higgins et al. 2023). A sensitivity analysis confirmed negligible impact of this correction on pooled estimates. Sensitivity analyses excluding studies with “some concerns” on risk of bias were also conducted. Potential small‐study effects were assessed using Egger's regression test (Lin and Chu 2018). Prediction intervals were calculated for primary outcomes using the method of Riley et al. (2011).

### 2.8 Certainty of Evidence

The certainty of the evidence for each outcome was assessed using the Grading of Recommendations Assessment, Development, and Evaluation (GRADE) approach. The quality of evidence was downgraded based on five domains: risk of bias, inconsistency, indirectness, imprecision, and publication bias. We summarized the findings in a GRADE Summary of Findings table to provide a transparent rating of our confidence in the pooled effect estimates (Guyatt et al. 2011).

## 3 Results

### 3.1 Overview of Study Identification and Screening Process

A total of 1608 records were initially retrieved from eight databases and four records retrieved from gray literature. After eliminating 814 duplicate entries, 798 records remained for title and abstract screening. Of these, 766 were excluded for failing to meet the selection criteria. This left 33 studies for full‐text review, of which 21 were excluded for various reasons (Supporting Information S1: Table 5). Ultimately, 12 publications from 12 randomized controlled trials were included in the review, and 12 trials were eligible for data pooling in the meta‐analysis (Figure 1).

**FIGURE 1:**
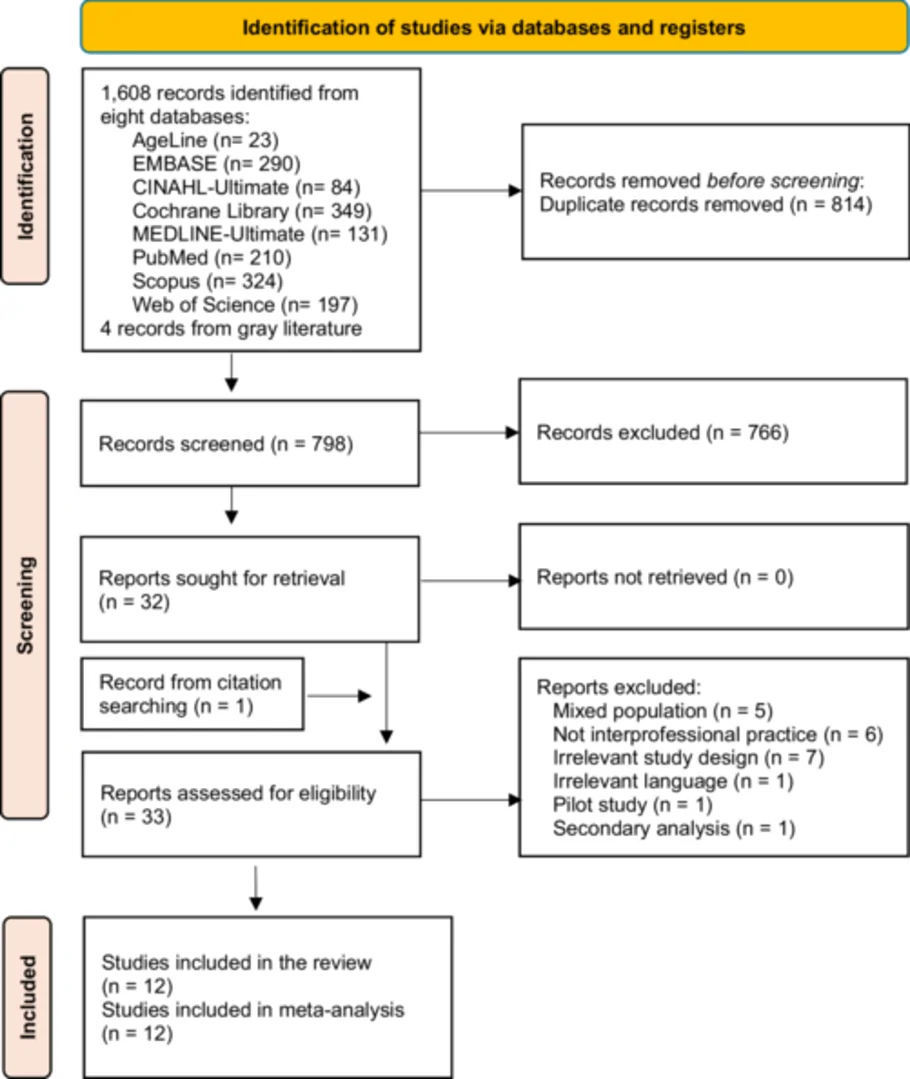
Flowchart of study selection.

### 3.2 Overview of Included Studies and Their Characteristics

Among the 12 included trials, two were conducted in Taiwan (Shyu et al. 2008; Shyu et al. 2016), two each in Australia (Crotty et al. 2019; Swanson et al. 1998), Sweden (Berggren et al. 2019; Karlsson et al. 2016), and one each in Belgium (Panella et al. 2018), Canada (Naglie et al. 2002), China (Wu et al. 2023), Norway (Prestmo et al. 2015), Singapore (Chong et al. 2013), and Spain (Vidán et al. 2005). These studies were published between 1998 and 2023, with sample sizes ranging from 71 to 504 participants. A total of 2511 participants with hip fractures were included in the final analysis (Supporting Information S1: Table 6).

The participants across the included studies were older adults with hip fractures, with mean ages ranging from approximately 76 to 88 years. All studies reported the control groups received usual care. The interventions were delivered by various configurations of interprofessional collaborative teams, typically including geriatricians, orthopedic surgeons, nurses, physiotherapists, occupational therapists, dietitians, and social workers. Most interventions were initiated during the hospitalization phase and extended into the post‐discharge recovery period, such as through in‐home rehabilitation or community‐based follow‐up. The duration of interventions across studies ranged from 0.5 to 24 months, with follow‐up assessments conducted at multiple time points.

As summarized in Supporting Information S1: Table 7, all interventions incorporated comprehensive geriatric assessment and physiotherapy/rehabilitation, while nearly all (*n* = 12) included structured discharge planning. Home or community‐based rehabilitation was provided in six studies. Early surgery and early mobilization were applied in five and four studies, respectively. Nutritional care was included in only one study, whereas social work involvement was reported in two studies and psychological support in one study. Overall, the number of focal areas ranged from 2 to 7, with nine of 12 studies (75%) addressing four or fewer areas. This variation highlights differences in team composition, healthcare contexts, and intervention priorities. Shyu et al. (2016) was a three‐arm trial; only the interdisciplinary care arm versus the control arm was extracted. An effective sample size was applied to Panella et al. (2018) using a design effect (DEFF = 1.342; ICC = 0.018; mean cluster size = 20), yielding effective ICP *n* = 224 and control *n* = 159.

Common outcomes included activity of daily living, length of stay, quality of life, and mortality. Activity of daily living was most frequently assessed using the Barthel Index, with only one study employing the Harris Hip Score. Quality of life was measured using validated instruments such as the 12‐item Short Form Health Survey (SF‐12), 36‐item Short Form Health Survey (SF‐36), and EuroQoL 5‐Dimensions (EQ‐5D; EQ‐5D‐3L in Prestmo et al. 2015; EQ‐5D‐5L in Crotty et al. 2019; version unspecified in Panella et al. 2018). Outcome reporting was also mapped against the Core Outcome Set (COS) recommended for hip fracture research, comprising mortality, pain, activities of daily living, mobility, and quality of life. Eleven trials reported activities of daily living, seven reported mortality, and five reported quality of life, whereas pain (two trials) and mobility (four trials) were less frequently addressed. Only three trials assessed four or more COS outcomes (Supporting Information S1: Table 8).

### 3.3 Risk of Bias in Studies

Five of the included studies were found to have a low risk of bias, six raised some concerns, and one (Wu et al. 2023) was rated high risk (Figure 2). Among the five domains assessed using the Cochrane Risk of Bias 2.0 tool, the most frequently identified issue was risk of bias arising from measurement of the outcome (five studies), followed by the randomization process (four studies) and deviations from the intended intervention (three studies); no studies showed concerns for missing outcome data, and one study showed concerns for selection of the reported result.

**FIGURE 2:**
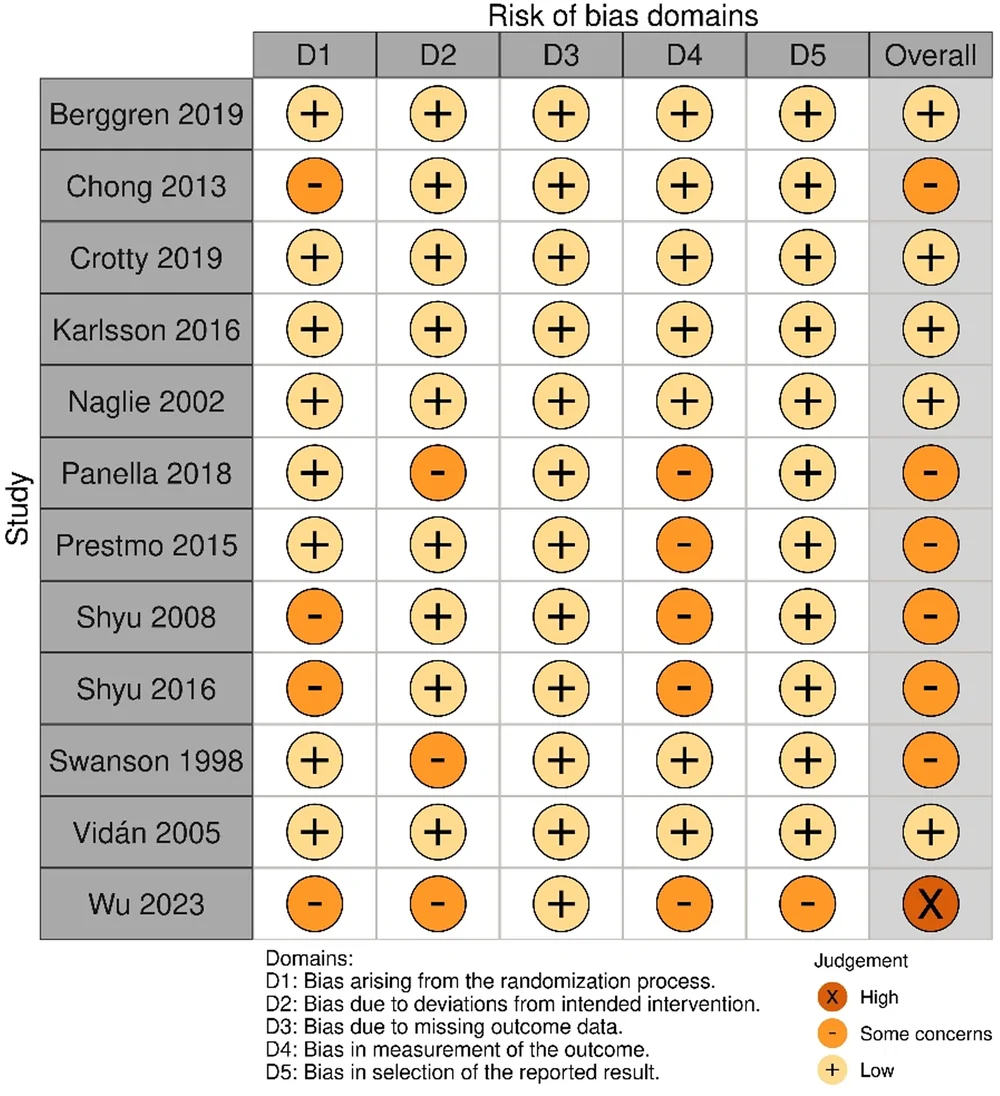
Risk of bias appraisal.

### 3.4 Activities of Daily Living

Four studies (*N* = 824; Chong et al. 2013; Prestmo et al. 2015; Shyu et al. 2008; Shyu et al. 2016) assessed activities of daily living within 6 months using the Barthel Index (0–100 scale). One trial (Prestmo et al. 2015) originally reported 0–20 scores that were rescaled ×5 prior to pooling. Under the random‐effects model, interprofessional collaborative practice significantly improved recovery compared with usual care (MD = +4.86 points, 95% CI [+3.71, +6.02]; *p* < 0.001). Heterogeneity was low (I^2^ = 9.7%, 95% CI [0.0%, 81.3%]; τ^2^ = 0.382). The 95% prediction interval [+1.19, +8.54 points] suggests the benefit would likely extend to future trials. An exploratory Egger's test showed no significant evidence of small‐study effects (intercept *p* = 0.752); given the small number of contributing studies (k = 4), this result should be interpreted cautiously (Figure 3a).

**FIGURE 3:**
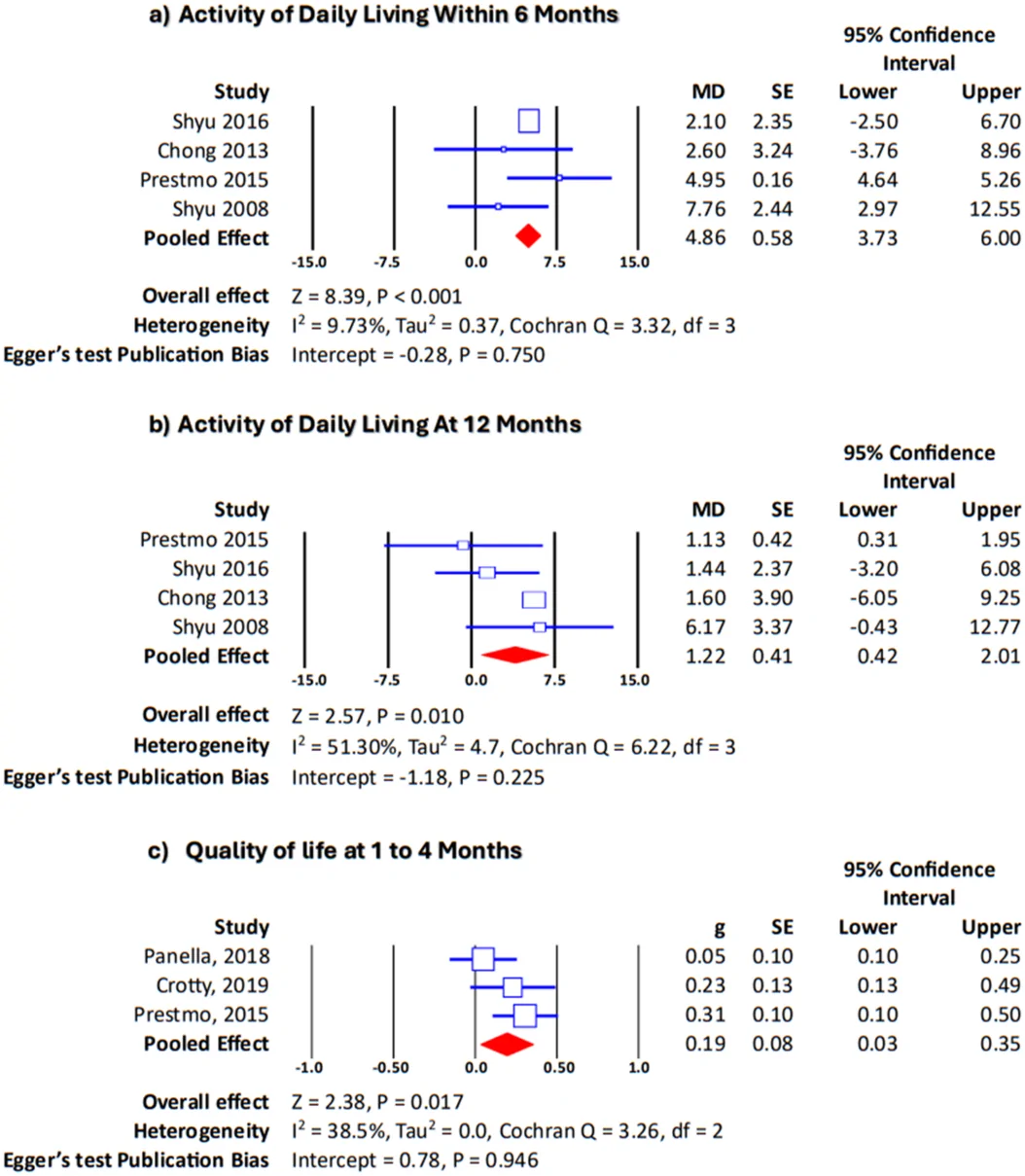
Forest plot for pooled effect size on (a) Activity of Daily Living within 6 months, (b) Activity of Daily Living at 12 months, (c) Quality of Life at 1–4 months. MD, mean difference; SE, standard error.

At 12 months, four studies (*N* = 783) continued to favor interprofessional collaborative practice (MD = +3.92 points, 95% CI [+0.90, +6.93]; *p* = 0.011). Heterogeneity was moderate (I^2^ = 51.7%, 95% CI [0.0%, 84.2%]; τ^2^ = 4.791). The 95% prediction interval [−7.59, +15.43 points] suggests the benefit may not consistently extend to future trials. Exploratory subgroup analyses found no significant moderator across setting, era, region, or risk of bias. Egger's test was not interpretable (k < 10) (Figure 3b).

### 3.5 Quality of Life

Three studies (*N* = 1013; Crotty et al. 2019; Panella et al. 2018; Prestmo et al. 2015) measured health‐related quality of life using the EQ‐5D at 1–4 months post‐discharge. Instrument versions differed across contributing studies (EQ‐5D‐3L: Prestmo et al. 2015; EQ‐5D‐5L: Crotty et al. 2019; version unspecified: Panella et al. 2018), representing a source of clinical heterogeneity. Under the random‐effects model the pooled standardized mean difference was Hedges’ G = +0.194 (95% CI [+0.034, +0.354]; *p* = 0.017; I^2^ = 38.6%; τ^2^ = 0.008), indicating a small‐to‐moderate improvement in the interprofessional collaborative practice group. A sensitivity analysis using REML with Hartung–Knapp correction yielded a non‐significant result (Hedges’ G = +0.194, 95% CI [−0.154, +0.541]; *p* = 0.183), confirming the result is not robust at k = 3. Egger's test was not interpretable (k < 10) (Figure 3c).

### 3.6 Length of Stay

Six studies (*N* = 1320; Berggren et al. 2019; Panella et al. 2018 [DEFF‐adjusted]; Shyu et al. 2008; Shyu et al. 2016; Vidán et al. 2005; Wu et al. 2023) showed that interprofessional collaborative practice significantly reduced hospital stay compared with usual care, with a pooled MD of −2.26 days (95% CI [−3.64, −0.88]; *p* = 0.001; I^2^ = 82.0%, 95% CI [61.6%, 91.5%]; τ^2^ = 2.330). The 95% prediction interval [−6.92, +2.41 days] suggests the reduction in length of stay may not hold across all future trial settings. No significant funnel plot asymmetry was detected by Egger's test (*p* = 0.979); nevertheless, the result remains exploratory given the modest number of pooled studies (k = 6) (Figure 4a). A series of exploratory subgroup and meta‐regression were performed to explore potential source of heterogeneity (Supporting Information S1: Table 9). An exploratory subgroup analysis by collaboration intensity showed a significant between‐subgroup difference: high‐intensity trials (≥ 5 focal areas) MD = −4.42 days (95% CI [−6.86, −1.98]) versus standard‐intensity trials (≤ 4 focal areas) MD = −1.26 days (95% CI [−2.27, −0.26]); Q(1) = 5.50, *p* = 0.019. Subgroup analyses by setting, era, region, and risk of bias showed no significant between‐subgroup differences. The Panella DEFF correction had negligible impact (Δ = 0.13 days) (Supporting Information S1: Table 10). An exploratory meta‐regression identified mean patient age as a significant predictor of LOS reduction (β = −0.447 days per year, 95% CI [−0.810, −0.083]; t[4] = −3.41; *p* = 0.027), accounting for 84.0% of between‐study variance (Supporting Information S1: Table 11).

**FIGURE 4:**
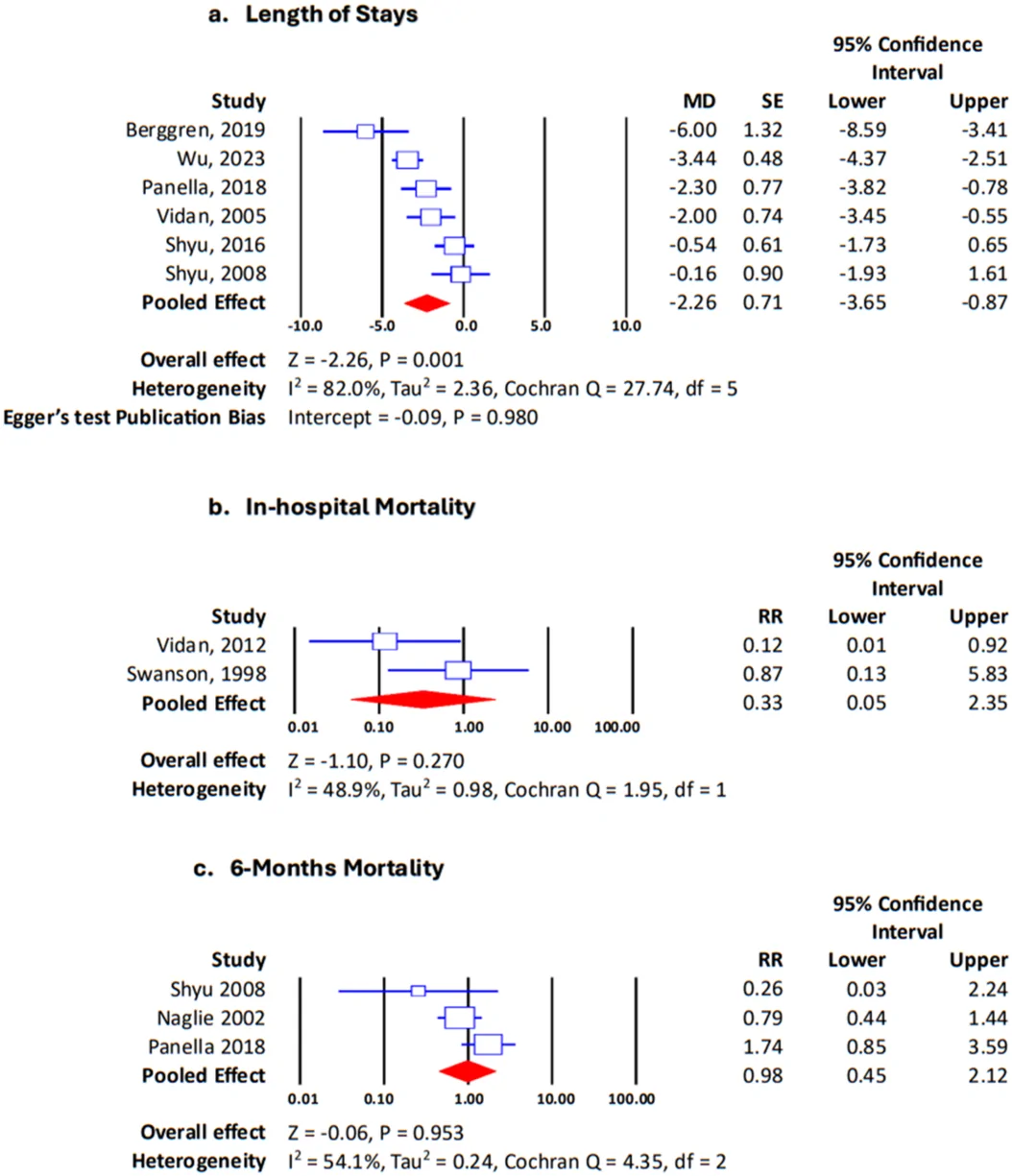
Forest plot for pooled effect size on (a) Length of Stay and (b) All‐cause Mortality. MD, mean difference; RR, risk ratio; SE, standard error.

### 3.7 Mortality

Five studies reported mortality at two follow‐up windows, in‐hospital and 6 months. Mortality was therefore analyzed separately by time point using a random‐effects model. For in‐hospital mortality (two studies, *n* = 390), interprofessional collaborative practice was not associated with a statistically significant reduction in mortality compared with usual care (RR = 0.33, 95% CI: 0.05–2.35; *p* = 0.270), with moderate heterogeneity (I^2^ = 48.90%) (Figure 4b). For 6‐month mortality (three studies, *N* = 803; Naglie et al. 2002; Panella et al. 2018; Shyu et al. 2008), there was also no statistically significant difference between groups (RR = 0.977, 95% CI [0.450, 2.119]; *p* = 0.953), and heterogeneity was substantial (I^2^ = 54.1%) (Figure 4c). Fixed‐effect sensitivity confirmed the conclusion (RR = 1.023, 95% CI [0.653, 1.604]; *p* = 0.920). Absolute risk difference: −0.22% (95% CI [−5.29%, +10.78%]); confidence interval spans both substantial benefit and potential harm, confirming inconclusive evidence. Given the small number of included studies in each time point analysis, small‐study effects and publication bias were not formally assessed. Fixed‐effect sensitivity for in‐hospital mortality: RR = 0.345 (95% CI [0.085, 1.393]; *p* = 0.135), consistent with the random‐effects result. Absolute risk difference for in‐hospital mortality: −3.73% (95% CI [−5.32%, +7.56%]); the confidence interval spans both substantial benefit and potential harm.

### 3.8 Certainty of Evidence

Using the GRADE approach, the certainty of evidence for the primary outcomes ranged from very low to low (Table 1). For activity of daily living within 6 months (k = 4, *N* = 824), certainty was rated Low (⊕⊕◯◯), downgraded for serious risk of bias (all four contributing trials rated “some concerns“) and serious imprecision (lower 95% CI bound of +3.71 points approaches the hip‐fracture‐specific MCID of 7.1 points; de Morton et al. 2013). For activity of daily living at 12 months (k = 4, *N* = 783), certainty was downgraded to Very Low (⊕◯◯◯) for serious risk of bias, serious inconsistency (I^2^ = 51.7%, unexplained by any tested moderator), and serious imprecision (lower CI bound +0.90 points, below the 12‐month MCID of 9.8 points; Unnanuntana et al. 2018). Certainty for quality of life (k = 3, *N* = 1013) was downgraded to Very Low (⊕◯◯◯) for serious risk of bias (two of three contributing trials rated “some concerns”), serious indirectness (EQ‐5D instrument version heterogeneity), and serious imprecision (REML+Hartung–Knapp: *p* = 0.183, non‐significant). For length of stay (k = 6, *N* = 1320), certainty was rated Low (⊕⊕◯◯), downgraded for serious risk of bias (including one contributing trial rated high risk) and serious inconsistency (I^2^ = 82.0%, rated Serious rather than Very Serious because exploratory meta‐regression showed mean patient age accounted for 84.0% of between‐study variance). For mortality, certainty was assessed separately by follow‐up period. For in‐hospital mortality (k = 2), certainty was Low (⊕⊕◯◯), downgraded for serious risk of bias and serious imprecision (95% CI [0.047, 2.354] spans large benefit to potential harm). For 6‐month mortality (k = 3), certainty was Very Low (⊕◯◯◯), downgraded for serious risk of bias (two of three contributing trials rated “some concerns”), serious inconsistency (I^2^ = 54.1%), and serious imprecision (95% CI [0.450, 2.119] crosses the null).

**TABLE 1:** GRADE evidence profile: interprofessional collaborative practice versus usual care for hip fracture in older adults (12 RCTs; *N* = 2511).

Outcome (follow‐up)	Studies (participants)	Certainty assessment domains					Overall certainty	Effect (95% CI)
		Risk of bias	Inconsistency	Indirectness	Imprecision	Publication bias		
ADL function ≤ 6 months	k = 4 *N* = 824 (ICP 414; Ctrl 410)	**Serious ↓**	Not serious	Not serious	**Serious ↓**	Undetected	**Low ⊕⊕◯◯**	MD +4.86 [+3.71, +6.02]^a,b^
ADL function 12 months	k = 4 *N* = 783 (ICP 404; Ctrl 379)	**Serious ↓**	**Serious ↓**	Not serious	**Serious ↓**	Undetected	**Very Low ⊕◯◯◯**	MD +3.92 [+0.90, +6.93]^a,b,c^
Quality of life (EQ‐5D)	k = 3 *N* = 1013 (ICP 537; Ctrl 476)	**Serious ↓**	Not serious	**Serious ↓**	**Serious ↓**	Undetected	**Very Low ⊕◯◯◯**	Hedges’ G +0.194 [+0.034, +0.354]^a,d,e^
Length of stay (days)	k = 6 *N* = 1320 (ICP 693; Ctrl 627)	**Serious ↓**	**Serious ↓**	Not serious	Not serious	Undetected	**Low ⊕⊕◯◯**	MD −2.26 [−3.64, −0.88]^a,f^
In‐hospital mortality	k = 2 *N* = 390 (ICP 193; Ctrl 197)	**Serious ↓**	Not serious	Not serious	**Serious ↓**	Undetected	**Low ⊕⊕◯◯**	RR 0.33 [0.05, 2.35] ARD −3.7% (CR = 5.6%)^a,g,h^
6‐month mortality	k = 3 *N* = 803 (ICP 450; Ctrl 353)	**Serious ↓**	**Serious ↓**	Not serious	**Serious ↓**	Undetected	**Very Low ⊕◯◯◯**	RR 0.98 [0.45, 2.12] ARD −0.2% (CR = 9.6%)^a,g,h,i^

*Note:* Grading criteria follow the GRADE Working Group (Guyatt et al. 2011). ⊕⊕⊕⊕ = High; ⊕⊕⊕◯ = Moderate; ⊕⊕◯◯ = Low; ⊕◯◯◯ = Very Low. MD = mean difference (positive values favor ICP); RR = risk ratio (values < 1 favor ICP); G = Hedges’ corrected standardized mean difference; ARD = absolute risk difference; CR = control group event rate. ICP = interprofessional collaborative practice; CI = confidence interval; RoB = risk of bias; FE = fixed‐effect model; REML + HK = restricted maximum likelihood with Hartung–Knapp adjustment. Shading: green = Moderate certainty; yellow = Low certainty; orange = Very Low certainty. ***a*** Risk of bias: Cochrane RoB 2.0 assessment identified five trials rated “some concerns” (Chong et al. 2013; Panella et al. 2018; Prestmo et al. 2015; Shyu et al. 2008; Shyu et al. 2016; Swanson et al. 1998) and one trial rated “high risk” (Wu et al. 2023), concentrated in the randomization process, deviations from intended interventions, and measurement‐of‐outcome domains. These trials contribute the majority or entirety of the pooled trials for activities of daily living (4/4), quality of life (2/3), length of stay (4/6, including the one high‐risk trial), 6‐month mortality (2/3), and in‐hospital mortality (1/2); risk of bias was therefore rated Serious for every outcome. Length of stay was not escalated to Very Serious despite the contributing high‐risk trial because it represents only one of six pooled trials. ***b*** Barthel Index scale comparability: Prestmo et al. (2015) used the 0–20 BI; all other trials used the 0–100 modified BI. Scores multiplied ×5 prior to pooling. This conversion assumes linearity across the scale range. Karlsson et al. (2016), which also used the 0–20 BI, was not included in this pool (see Methods 2.7). ***c*** Inconsistency: ADL 12 m I^2^ = 51.7% not explained by setting, era, region, geriatrician involvement, risk of bias, or collaboration intensity subgroup analyses. Unexplained heterogeneity contributes to downgrading. ***d*** Indirectness: Prestmo et al. (2015) used EQ‐5D‐3L with UK time‐trade‐off value set; other trials’ value set versions unconfirmed. Country‐specific value sets introduce non‐comparability in utility scores. ***e*** REML + HK sensitivity: DerSimonian–Laird model (G = +0.194, *p* = 0.017) does not replicate under REML with Hartung–Knapp correction (G = +0.194, 95% CI [−0.154, +0.541], *p* = 0.183). Statistical significance of QoL benefit is not robust to this correction. ***f*** Inconsistency: LOS I^2^ = 82.0% rated Serious (not Very Serious) because exploratory meta‐regression showed mean patient age accounts for 84.0% of between‐study variance in LOS (β_1_ = −0.447 days/year, 95% CI [ − 0.810, −0.083], *p* = 0.027; k = 6). Residual heterogeneity after adjusting for age was non‐significant: Q_res(4) = 4.98, *p* = 0.290. ***g*** Imprecision: confidence intervals for both mortality outcomes span clinically important benefit and harm (in‐hospital mortality 95% CI [0.05, 2.35]; 6‐month mortality 95% CI [0.45, 2.12]), with few contributing studies at each time point; imprecision was rated Serious for both outcomes. ***h*** Fixed‐effect sensitivity: In‐hospital mortality RR = 0.345 [0.085, 1.393], *p* = 0.135; 6‐month mortality RR = 1.023 [0.653, 1.604], *p* = 0.920. Both outcomes remain non‐significant and conclusions are unchanged. ***i*** 6‐month mortality: Panella et al. (2018) is directionally inconsistent with Naglie et al. (2002) and Shyu et al. (2008): RR = 1.74 versus 0.79 and 0.26 respectively. Panella's pathway model and cluster RCT design may contribute to this divergence.

## 4 Discussion

### 4.1 Summary of Main Findings

This systematic review and meta‐analysis synthesized evidence from randomized controlled trials on interprofessional collaborative practice for older adults with hip fractures. Interprofessional models were associated with improved activities of daily living, modest improvements in quality of life, and shorter hospital length of stay compared with usual care, with certainty ranging from very low to low across outcomes. In contrast, mortality evidence was inconclusive. Time‐point‐specific analyses found no statistically significant difference in in‐hospital or 6‐month mortality, and confidence intervals were wide, spanning both potential benefit and potential harm; results should be interpreted as inconclusive rather than as evidence of no effect. The certainty of evidence was low for activities of daily living within 6 months, length of stay, and in‐hospital mortality and very low for activities of daily living at 12 months, quality of life, and 6‐months mortality outcomes.

### 4.2 Outcomes of Interprofessional Collaborative Practice

Interprofessional collaborative practice improved recovery in activity of daily living at both short and longer follow‐up. Within 6 months, coordinated rehabilitation, early mobilization, and individualized goal setting yielded a meaningful functional advantage. Benefits persisted for 12 months, suggesting effects beyond the immediate post‐acute phase. Shared decision‐making among geriatricians, physiotherapists, nurses, and other disciplines likely supports tailored recovery and earlier restoration of independence. Hip‐fracture‐specific minimal clinically important differences (MCIDs) for the Barthel Index have been established at 7.1 points at the inpatient rehabilitation stage (de Morton et al. 2013) and at 9.8 points at 12‐month follow‐up (Unnanuntana et al. 2018). The pooled mean differences of +4.86 points at 6 months and +3.92 points at 12 months were statistically significant but fell below both thresholds, indicating that while interprofessional collaborative practice consistently improved recovery at the population level, the average effect may not reach the threshold perceptible to individual patients. These findings align with broader evidence that coordinated, multidisciplinary care improves functional outcomes, in comprehensive geriatric assessment (Ellis et al. 2011), organized stroke units (Langhorne et al. 2020), and orthogeriatric co‐management (Grigoryan et al. 2014). In contrast, conventional care often delays mobilization and lacks continuity between disciplines, whereas interprofessional pathways integrate assessment, therapy, and review to support day‐to‐day reintegration.

The pooled reduction in length of hospital stays, averaging just over 2 days, reflects a clinically meaningful improvement that may ease healthcare system burdens and reduce patient exposure to hospital‐acquired complications. Similar LOS reductions have been reported in observational orthogeriatric co‐management studies (Grigoryan et al. 2014) and enhanced recovery pathway reviews (Liu et al. 2021). The streamlined coordination of care under interprofessional collaborative practice through early surgical consultation, prompt rehabilitation, and proactive discharge planning likely explains this outcome. Exploratory meta‐regression identified mean patient age as a significant predictor of length‐of‐stay benefit; this age‐related pattern suggests interprofessional coordination may benefit older, frailer patients most, warranting stratified analysis in future trials. Unlike traditional models where fragmented communication causes care delays, interprofessional collaborative practice facilitates more efficient transitions across care milestones, thereby shortening hospitalization without compromising safety.

This review also found that interprofessional collaborative practice had a positive effect on quality of life, especially in domains such as mobility, emotional well‐being, and self‐care. Although the pooled effect was modest, the improvement is noteworthy given the complexity of recovery in this population and the multifactorial influences on quality of life. These improvements are likely due to the holistic nature of interprofessional collaborative practice, which addresses not only physical rehabilitation but also psychosocial support, caregiver involvement, and post‐discharge continuity. Similar quality‐of‐life gains have been reported with coordinated stroke unit care and integrated chronic disease‐management programs (Kruis et al. 2013). Although a few trials reported neutral findings, these may be attributed to brief intervention duration or limited follow‐up time insufficient to capture long‐term quality of life trajectories. The statistical significance of the pooled quality‐of‐life result was not robust to the Hartung–Knapp sensitivity analysis and should therefore be interpreted with caution given only three contributing trials with heterogeneous EQ‐5D instrument versions.

Mortality evidence was inconclusive in both time‐point‐specific analyses. For in‐hospital mortality, the point estimate suggested a possible reduction in deaths, but the confidence interval was very wide, indicating substantial uncertainty about the magnitude and direction of any effect. Fixed‐effect sensitivity yielded a consistent conclusion. For 6‐month mortality, the RR was close to unity, with absolute risk difference of −0.22% (95% CI [−5.29%, +10.78%]) spanning both benefit and harm. These confidence intervals should be interpreted as inconclusive evidence rather than evidence of no effect. Differences in intervention fidelity, baseline risk, and comparator contamination with informal co‐management may partly explain the lack of a detectable mortality signal; larger, adequately powered trials are needed to clarify any effect on survival. Observational evidence suggests possible survival benefits of coordinated geriatric care (Ellis et al. 2011; Grigoryan et al. 2014), though this remains unconfirmed by RCT‐level data. Taken together, our findings suggest that any survival benefit of interprofessional collaborative practice may be context‐dependent and difficult to detect without adequately powered trials, standardized mortality time points, and clearer reporting of co‐management practices within the comparator condition. The evolving nature of usual care across the 1998–2023 trial span is a further contextual factor that may contribute to between‐study heterogeneity across all outcomes and limit direct comparability of effect sizes across trials.

### 4.3 Outcome Reporting Against the Hip Fracture Core Outcome Set

Over a decade ago, a core outcome set for hip fracture research was developed to standardize outcome reporting and ensure that studies address domains most relevant to patients and clinicians (Haywood et al. 2014). The hip fracture core outcome set recommends the inclusion of mortality, pain, activities of daily living, mobility, and quality of life. In this review, inconsistent use of the core outcome set was observed. This was expected, as 46% of included trials predated the core outcome set's introduction, with others likely recruiting beforehand. This mirrors low core outcome set uptake reported elsewhere, including 98% non‐adoption across 95 high‐impact trials (Matvienko‐Sikar et al. 2022), attributable in part to barriers in design and perceived relevance (Hughes et al. 2022; Smith et al. 2019). Greater promotion and stronger incentives for core outcome set use are needed to improve consistency in hip fracture research.

### 4.4 Implication to Clinical Practice

Implementing interprofessional collaborative practice from admission through post‐discharge follow‐up remains clinically valuable, targeting mechanisms that plausibly reduce preventable complications: early geriatric assessment, proactive monitoring, timely management of deterioration, medication safety, and coordinated rehabilitation. Effects on activities of daily living and length of stay were the most consistent findings, though local implementation and context likely shape impact, and any mortality benefit remains inconclusive. Nurses occupy a pivotal role within interprofessional collaborative practice, having been part of the team in all 12 included trials. As the clinical team members with the most continuous patient contact, nurses are uniquely positioned to coordinate across disciplines, monitor for early deterioration, manage pain and delirium, facilitate mobilization, and ensure continuity of care, including via nurse‐led assessment by advanced practice nurses in orthogeriatric models. Nursing‐sensitive outcomes likely to benefit include functional recovery, complication prevention (delirium, falls, pressure injury), medication reconciliation, and discharge readiness. Future trials should report nursing‐specific contributions and include such outcomes as primary endpoints. Healthcare systems should prioritize interprofessional training, institutional support for collaborative protocols with defined nursing coordination roles, and infrastructure enabling coordinated discharge planning across settings. Embedding these principles across the care continuum may optimize recovery and continuity, while future trials clarify whether gains extend to survival.

### 4.5 Limitation

This review has methodological limitations. First, restricting inclusion to English‐language publications may have introduced language bias and reduced representativeness. Trial registries were not searched independently; however, the Cochrane Library search (349 records) included CENTRAL, which indexes ClinicalTrials.gov and the WHO International Clinical Trials Registry Platform, capturing any registered trials. Trials evaluating enhanced recovery after surgery pathways were excluded to maintain a clear focus on interprofessional collaborative practice; this improves conceptual coherence but may limit generalizability given overlapping elements with team‐based care. The quality‐of‐life synthesis was limited by EQ‐5D instrument version heterogeneity (EQ‐5D‐3L: Prestmo et al. 2015; EQ‐5D‐5L: Crotty et al. 2019; version unspecified: Panella et al. 2018); differing value sets may affect comparability of utility scores across studies. Several meta‐analyses were based on few contributing trials, reducing precision; Egger's regression test for publication bias was underpowered (k < 10 for all outcomes), so funnel plots are presented for visual inspection only and should not be interpreted as definitive evidence of publication bias. The usual care comparator varied across trials and evolved over the 1998–2023 span; several control arms may have incorporated informal multidisciplinary elements, potentially attenuating the measured ICP effect, a recognized limitation of pragmatic RCTs that should be considered when interpreting pooled estimates. Finally, mapping to the hip fracture core outcome set and coding of intervention focal areas relied on primary trial reporting, which was sometimes incomplete; residual classification error is possible.

## 5 Conclusion

This systematic review and meta‐analysis suggests that interprofessional collaborative practice, implemented from hospital admission through post‐discharge follow‐up, is associated with improved activities of daily living, modest quality‐of‐life gains, and shorter hospital stays among older adults with hip fractures. Mortality effects were inconclusive at in‐hospital and 6‐month follow‐up. These findings support coordinated, interprofessional approaches addressing both acute care needs and functional recovery, with nurses playing key roles in coordination, mobilization, discharge planning, and continuity of care. Given the predominantly low certainty of evidence, these findings remain provisional; cautious integration of interprofessional elements may be reasonable while future, adequately powered trials clarify outcome time points and usual care co‐management practices to determine survival effects.

## Author Contributions

**Santo Imanuel Tonapa:** conceptualization, methodology, data curation, investigation, writing – original draft preparation, writing – review and editing. **I‐Chin Huang:** data curation, investigation, writing – original draft preparation, writing – review and editing. **Bih‐O Lee:** conceptualization, methodology, data curation, investigation, writing – original draft preparation, writing‐review and editing. **Wendy Chaboyer:** conceptualization, methodology, validation, visualization, supervision, writing – review and editing.

## Funding

This study did not receive any specific grant from funding agencies in the public, commercial, or non‐profit sectors.

## Conflicts of Interest

The authors declare no conflicts of interest.

## Supporting information


**Table S1:** Collaborative Mechanisms Across Included Trials: ICP Classification. **Table S2:** Usual Care Characteristics Across Included Trials. **Table S3:** Conceptual Classification of Included Trials: Interprofessional Collaborative Practice, Comprehensive Geriatric Assessment, and Orthogeriatric Co‐management. **Table S4:** Search strategy. **Table S5:** Reasons Studies were Excluded During Full‐Text Screening (n = 21). **Table S6:** Characteristics of Included Trials. **Table 7:** Interprofessional Collaborative Practice Focus Areas Across Studies. **Table S8:** Use of the Hip Fracture Core Outcome Set. **Table S9:** Conceptual and Operational Definitions of All Moderators Used in Subgroup and Meta‐Regression Analyses. **Table S10:** Exploratory Subgroup Analyses for All Outcomes: Interprofessional Collaborative Practice Versus Usual Care in Hip Fracture. **Table S11:** Exploratory Meta‐Regression Analyses for Length of Stay (k = 6): Interprofessional Collaborative Practice Versus Usual Care in Hip Fracture.

## Data Availability

Data sharing not applicable to this article as no datasets were generated or analyzed during the current study.
