# Supplementary material for: Effects of Interprofessional Collaborative Practice in Older Adults’ Hip Fractures Care: A Systematic Review and Meta‐Analysis of Randomized Controlled Trials
Source: Res Nurs Health. 2026 Sep 8;49(6):909–21. doi: 10.1002/nur.70125 (PMC13729724; doi:10.1002/nur.70125)
Supplement: Supplementary file 1 [file NUR-49-909-s001.docx]

**Supplementary File**

- **Supplementary Table 1.** Collaborative Mechanisms Across Included Trials: ICP Classification.
- **Supplementary Table 2.** Usual Care Characteristics Across Included Trials
- **Supplementary Table 3.** Conceptual Classification of Included Trials: Interprofessional Collaborative Practice, Comprehensive Geriatric Assessment, and Orthogeriatric Co-management.
- **Supplementary Table 4.** Search strategy.
- **Supplementary Table 5.** Reasons Studies were Excluded During Full-Text Screening (n = 21).
- **Supplementary Table 6**. Characteristics of Included Trials.
- **Supplementary Table 7.** Interprofessional Collaborative Practice Focus Areas Across Studies.
- **Supplementary Table 8.** Use of the Hip Fracture Core Outcome Set.
- **Supplementary Table 9.** Conceptual and Operational Definitions of All Moderators Used in Subgroup and Meta-Regression Analyses.
- **Supplementary Table 10.** Exploratory Subgroup Analyses for All Outcomes: Interprofessional Collaborative Practice Versus Usual Care in Hip Fracture.
- **Supplementary Table 11.** Exploratory Meta-Regression Analyses for Length of Stay (k = 6): Interprofessional Collaborative Practice Versus Usual Care in Hip Fracture.

**Supplementary Table 1**. Collaborative Mechanisms Across Included Trials: ICP Classification

| **Study** | **Country** | **Minimum criteria for ICP classification** | | | | **Collaborative mechanisms present** | | | | **Classification** | **Basis for classification** |
| --- | --- | --- | --- | --- | --- | --- | --- | --- | --- | --- | --- |
|  |  | **C1 ≥3 disciplines** | **C2 Shared care plan** | **C3 Structured joint decision-making** | **C4 Multi-phase coordination** | **M1 Shared/joint care plan** | **M2 Scheduled joint rounds/meetings** | **M3 Integrated documentation** | **M4 Designated coordinator** |  |  |
| Berggren 2019 | Sweden | ✓ | ✓ | ✓ | ✓ | ✓ | ✓ | △ | ✓ | MEETS | Companion publication to Karlsson et al. (2016) — pre-planned secondary analysis reporting complications and readmissions from the same trial (ISRCTN 15738119).  Geriatric interdisciplinary team (geriatrician, physiotherapist, occupational therapist, nurse).  Individually designed home rehabilitation programme; geriatric ward rounds; dedicated geriatric team as coordinator; post-discharge follow-up. |
| Chong 2013 | Singapore | ✓ | △ | △ | ✓ | △ | △ | ✓ | △ | BORDERLINE | Protocol-driven integrated care pathway (not an individualised per-patient plan).  Three disciplines (physician, physiotherapist, nurse); structured protocol with documentation; no explicit interdisciplinary rounds reported.  Retained on the basis that the ICP label and multi-professional coordination meet the minimum threshold; flagged for sensitivity analysis. |
| Crotty 2019 | Australia | ✓ | ✓ | ✓ | ✓ | ✓ | ✓ | △ | ✓ | MEETS | Hospital outreach team delivering comprehensive geriatric assessment plus an individualised care plan.  Geriatrician, physiotherapist, dietitian, nurse; geriatrician met with families for progress review.  Post-discharge outreach to nursing care facility. |
| Karlsson 2016 | Sweden | ✓ | ✓ | ✓ | ✓ | ✓ | ✓ | △ | ✓ | MEETS | Primary trial publication (ISRCTN 15738119); companion publication: Berggren et al. (2019), pre-planned secondary analysis of complications and readmissions.  Geriatric interdisciplinary team (geriatrician, physiotherapist, occupational therapist, nurse).  Individually designed home rehabilitation programme; geriatric ward rounds; dedicated geriatric team as coordinator; post-discharge follow-up. |
| Naglie 2002 | Canada | ✓ | ✓ | ✓ | ✓ | ✓ | ✓ | ✓ | ✓ | MEETS | Strongest ICP example in the review. Internist-geriatrician, physiotherapist, occupational therapist, social worker, clinical nurse specialist.  Twice-weekly interdisciplinary rounds to set goals; documented shared care plan.  Care from admission through discharge planning. |
| Panella 2018 | Belgium | ✓ | △ | △ | ✓ | △ | △ | ✓ | △ | BORDERLINE | Hospital-level clinical pathway (7-phase protocol); cluster randomized design across 26 hospitals.  Surgeon, anaesthesiologist, physiotherapist, nurse; protocol adherence rather than individualised collaborative decision-making.  Geriatrician was rarely an integral team member (paper's own words). Retained because the pathway operationalises guideline-based interprofessional coordination across phases; flagged as closer to protocol-driven than decision-making ICP. |
| Prestmo 2015 | Norway | ✓ | ✓ | ✓ | ✓ | ✓ | ✓ | ✓ | ✓ | MEETS | Comprehensive geriatric care unit. Geriatrician, orthopaedic surgeon, nurse, physiotherapist, occupational therapist.  Structured systematic interdisciplinary comprehensive geriatric assessment; individualised rehabilitation plans.  Early discharge planning; inpatient and post-discharge phases. |
| Shyu 2008 | Taiwan | ✓ | ✓ | ✓ | ✓ | ✓ | ✓ | △ | ✓ | MEETS | Primary full-trial publication for Cohort A (n = 162; recruitment September 2001–November 2004).  Geriatrician, physiotherapist, nurse; in-hospital rehabilitation plus structured home visits.  Regular coordinated assessments; discharge planning included. |
| Shyu 2016 | Taiwan | ✓ | ✓ | ✓ | ✓ | ✓ | ✓ | △ | ✓ | MEETS | Independent Cohort B (n = 200; recruitment September 2005–July 2010) — no participant overlap with Cohort A (Shyu 2008).  Geriatrician, physiotherapist, nurse; enhanced interdisciplinary care arm extracted (three-arm trial).  Individualised care with weekly then biweekly home visits; discharge planning. |
| Swanson 1998 | Australia | ✓ | ✓ | ✓ | ✓ | ✓ | ✓ | △ | ✓ | MEETS | Geriatrician, physiotherapist, social worker, nurse, occupational therapist.  Early intensive interdisciplinary programme; trial coordinator for cross-disciplinary accountability.  Weekly case conferences; daily monitoring; 6-month follow-up. |
| Vidán 2005 | Spain | ✓ | ✓ | ✓ | ✓ | ✓ | ✓ | ✓ | ✓ | MEETS | Exemplary ICP structure. Geriatrician (daily), rehabilitation specialist, social worker, nurse.  Interdisciplinary meeting within 72 hours of admission to elaborate a comprehensive therapeutic plan; plan reviewed weekly.  Multi-professional accountability across the acute inpatient phase. |
| Wu 2023 | China | ✓ | ✓ | ✓ | ✓ | ✓ | ✓ | ✓ | ✓ | MEETS | Multidisciplinary team from 9 disciplines, 18 specialists.  Joint evaluation; jointly formulated treatment and rehabilitation plan; real-time coordination via WeChat.  Perioperative plus 12-month follow-up; the most interprofessionally intensive trial in the review. |

**Note.** Table includes all 12 publications retained in the final review following exclusion of Shyu et al. (2005; pilot study) and Tseng et al. (2012; secondary analysis with no extractable primary outcome data). ✓ = criterion/mechanism confirmed from primary paper; △ = partially present or ambiguous evidence; — = absent or not reported. Classification: MEETS = satisfies all four minimum criteria; BORDERLINE = satisfies ≥3 criteria but shared care plan (C2) or structured joint decision-making (C3) is protocol-driven rather than individualised collaborative; DOES NOT MEET = fails to satisfy ≥2 criteria. Studies classified as BORDERLINE are retained on the grounds that the intervention operationalises multi-professional coordination consistent with the WHO ICP framework, but are flagged for sensitivity analysis. Berggren et al. (2019) and Karlsson et al. (2016) are two independent publications from a single Swedish trial (ISRCTN 15738119); both are listed as separate rows as each is independently included in the review and contributes to the evidence base, though they report the same underlying intervention.

**Criteria:** (C1) Three or more professional disciplines involved; (C2) Shared, jointly developed care plan rather than parallel discipline-specific plans; (C3) At least one structured mechanism for collaborative decision-making (e.g., scheduled interdisciplinary team meetings or joint rounds); (C4) Coordination extending across more than one phase of the care pathway (e.g., acute inpatient care and post-discharge follow-up).

**Mechanisms:** (M1) Shared/joint care plan documented across disciplines; (M2) Scheduled interdisciplinary meetings, ward rounds, or case conferences; (M3) Integrated documentation system (e.g., shared records, WeChat coordination); (M4) Designated coordinator with cross-disciplinary accountability.

ICP = interprofessional collaborative practice; CGA = comprehensive geriatric assessment; WHO = World Health Organization.

**Supplementary Table 2.** Usual Care Characteristics Across Included Trials

| **Study (Country)** | **Usual Care Setting** | **Disciplines Routinely Involved** | **Informal Co-management Elements** |
| --- | --- | --- | --- |
| Berggren 2019 (Sweden) | Standard orthopaedic ward | Orthopaedic surgeon, ward nursing; physiotherapy by routine referral | None reported as standard; geriatric input not routinely available |
| Chong 2013 (Singapore) | Standard orthopaedic ward | Orthopaedic surgeon, ward nursing; physiotherapy by routine referral | Ad hoc physician consultation only; no structured geriatric pathway |
| Crotty 2019 (Australia) | Standard discharge pathway without outreach follow-up | Orthopaedic team, ward nursing, inpatient physiotherapy | Limited — no structured post-discharge geriatric or allied health outreach |
| Karlsson 2016 (Sweden) | Standard orthopaedic ward | Orthopaedic surgeon, ward nursing; physiotherapy by routine referral | None reported as standard; geriatric input not routinely available |
| Naglie 2002 (Canada) | Standard orthopaedic ward (non-geriatric unit) | Orthopaedic surgeon, ward nursing; physiotherapy and social work by as-needed referral | Geriatric consultation available on request only, not routine or proactive |
| Panella 2018 (Belgium) | Pre-existing hospital care pathways (pre-implementation standard) | Surgeon, anaesthesiologist, ward nursing | Varied by hospital; no standardised interprofessional coordination protocol |
| Prestmo 2015 (Norway) | Standard orthopaedic ward | Orthopaedic surgeon, ward nursing; physiotherapy by routine referral | None — explicitly compared against orthopaedic ward without geriatric co-management |
| Shyu 2008 (Taiwan) | Standard discharge planning without structured follow-up | Orthopaedic surgeon, ward nursing; physiotherapy by routine referral | Routine discharge instructions only; no structured home visit programme |
| Shyu 2016 (Taiwan) | Standard discharge planning without structured follow-up | Orthopaedic surgeon, ward nursing; physiotherapy by routine referral | Routine discharge instructions only; no structured home visit programme |
| Swanson 1998 (Australia) | Standard orthopaedic ward | Orthopaedic surgeon, ward nursing; physiotherapy and social work by as-needed referral | No dedicated case conferencing or cross-disciplinary coordinator |
| Vidán 2005 (Spain) | Standard orthopaedic department care | Orthopaedic surgeon, ward nursing; physiotherapy by routine referral | No daily geriatrician involvement; consultation available but not proactive |
| Wu 2023 (China) | Standard single-discipline orthopaedic care | Orthopaedic surgeon, ward nursing; physiotherapy by routine referral | No multidisciplinary team structure or shared documentation system |

**Note.** Usual care descriptions were synthesised from the care model reported in each primary trial publication.

**Supplementary Table 3.** Conceptual Classification of Included Trials: Interprofessional Collaborative Practice, Comprehensive Geriatric Assessment, and Orthogeriatric Co-management

| **Study** | **Contains CGA** | **Contains orthogeriatric co-management** | **Classified as ICP** | **Primary model** | **What distinguishes ICP from CGA or co-management alone** | **Setting and care phases** |
| --- | --- | --- | --- | --- | --- | --- |
| Berggren et al., 2019 (Sweden) | Yes | Yes | Yes | ICP with CGA | Pre-planned secondary analysis (complications and readmissions) of the same trial as Karlsson et al. (2016); team rounds + jointly developed goals across disciplines + post-discharge coordination distinguish from CGA alone | Inpatient + home (10 weeks) |
| Chong et al., 2013 (Singapore) | Yes | No | Yes | ICP — pathway model | Multi-professional pathway coordination across admission, rehabilitation, and discharge; shared protocol | Subacute rehabilitation |
| Crotty et al., 2019 (Australia) | Yes | No | Yes | ICP with CGA | Crosses institutional boundary; joint care plan with fortnightly interprofessional review | Post-acute (nursing care facility) |
| Karlsson et al., 2016 (Sweden) | Yes | Yes | Yes | ICP with CGA | Primary trial publication (ISRCTN 15738119); team rounds + jointly developed goals across disciplines + post-discharge coordination distinguish from CGA alone | Inpatient + home (10 weeks) |
| Naglie et al., 2002 (Canada) | Yes | Yes | Yes | ICP with CGA + co-management | Twice-weekly interdisciplinary rounds for goal-setting and monitoring; geriatrician co-manages with orthopaedics | Inpatient |
| Panella et al., 2018 (Belgium/Italy/Portugal) | Yes | No | Partial | ICP — pathway model (borderline) | Designated CP facilitator and clinical champions provide institutional collaborative mechanism; patient-level shared plan absent | Inpatient + 30 days |
| Prestmo et al., 2015 (Norway) | Yes | Yes | Yes | ICP with CGA + co-management | Geriatric care unit; daily team rounds; individualised plans developed and revised across all disciplines | Inpatient + early discharge |
| Shyu et al., 2008 (Taiwan) | Yes | No | Yes | ICP with CGA | Primary full-trial publication for Cohort A (n = 162); jointly developed rehabilitation goals; geriatric nurse-led coordination with physician and physiotherapist | Inpatient + home (3 months) |
| Shyu et al., 2016 (Taiwan) | Yes | No | Yes | ICP with CGA | Weekly interprofessional coordination; individualised rehabilitation revised over 4–12 months | Inpatient + home (12 months) |
| Swanson et al., 1998 (Australia) | Yes | Yes | Yes | Full ICP | Daily ward rounds + weekly case conferences + social work + home assessment; broadest phase coverage | Inpatient + home (6 months) |
| Vidán et al., 2005 (Spain) | Yes | Yes | Yes | ICP with CGA + co-management | Plan developed within 72 h in interdisciplinary meeting; weekly reviews; geriatrician and rehabilitation specialist jointly accountable | Inpatient (acute phase) |
| Wu et al., 2023 (China) | Yes | Yes | Yes | Full ICP | 18 specialists jointly formulate plan; real-time WeChat coordination; post-discharge remote follow-up | Inpatient + remote (12 months) |

**Note.** Three overlapping models are distinguished: comprehensive geriatric assessment (CGA) = structured multidimensional assessment process; orthogeriatric co-management = formal shared clinical responsibility between geriatrician and orthopaedic surgeon; interprofessional collaborative practice (ICP) = additionally requires structured collaborative decision-making across disciplines throughout the care continuum with a jointly developed and revised shared care plan. A study may contain elements of all three models; the primary label reflects the dominant organising structure. Panella et al. (2018) is classified as partial ICP because a standardised clinical pathway was implemented rather than an individualised jointly developed plan; retained with this qualification. Karlsson et al. (2016) and Berggren et al. (2019) are two independent publications from a single Swedish trial (ISRCTN 15738119); both are listed as separate rows. Shyu et al. (2008) reports the primary Cohort A trial (n = 162); Shyu et al. (2016) is an independent Cohort B trial with no participant overlap. CGA = comprehensive geriatric assessment; CP = care pathway; ICP = interprofessional collaborative practice.

# **Supplementary Table 4.** Search strategy

| **Databases** | **Set** | **Search terms** | **Record found** |
| --- | --- | --- | --- |
| **PubMed** | 1. | ( "older adults" OR "elderly" OR "geriatric" OR "aging" OR "senior" OR "aged"[MeSH Terms] ) AND ( "hip fracture" OR "hip fractures" OR "hip surgery" OR "hip injury" OR "hip fractures"[MeSH Terms] ) | 6,786,514 |
|  | 2. | ( "integrated care" OR "collaboration" OR "multi-disciplinary" OR "integrated approach" OR "interdisciplinary" OR "comprehensive" OR team* OR cooperat* OR collaborat* OR transprofession* OR transdisciplinar* OR multiprofession* OR multidisciplinar* OR interprofession* OR interdisciplinar* OR orthogeriatr* OR co-manag* OR "interprofessional relations"[MeSH Terms] OR "patient care team"[MeSH Terms] OR "intersectoral collaboration"[MeSH Terms]) | 2,115,278 |
|  | 3. | ( "RCT" OR "randomized controlled trial" OR "randomized controlled study" OR "randomly assigned" ) | 30,332 |
|  | **Total** | 1 AND 2 AND 3 | 210 |
| **AgeLine** | 1. | ("older adults" OR "elderly" OR "geriatric" OR "aging" OR "senior" OR (MH "Aged")) AND ("hip fracture" OR "hip fractures" OR "hip surgery" OR "hip injury" OR (MH "Hip Fractures")) | 1,380 |
|  | 2. | ("integrated care" OR "collaboration" OR "multi-disciplinary" OR "integrated approach" OR "interdisciplinary" OR "comprehensive" OR team* OR cooperat* OR collaborat* OR transprofession* OR transdisciplinar* OR multiprofession* OR multidisciplinar* OR interprofession* OR interdisciplinar* OR orthogeriatr* OR co-manag* OR (MH "Interprofessional Relations") OR (MH "Patient Care Team") OR (MH "Intersectoral Collaboration")) | 26,171 |
|  | 3. | ("RCT" OR "randomized controlled trial" OR "randomized controlled study" OR "randomly assigned" OR (MH "Randomized Controlled Trials")) | 5,441 |
|  | **Total** | 1 AND 2 AND 3 | 23 |
| **MEDLINE Ultimate** | 1. | ("older adults" OR "elderly" OR "geriatric" OR "aging" OR "senior" OR (MH "Aged")) AND ("hip fracture" OR "hip fractures" OR "hip surgery" OR "hip injury" OR (MH "Hip Fractures")) | 22,351 |
|  | 2. | ("integrated care" OR "collaboration" OR "multi-disciplinary" OR "integrated approach" OR "interdisciplinary" OR "comprehensive" OR team* OR cooperat* OR collaborat* OR transprofession* OR transdisciplinar* OR multiprofession* OR multidisciplinar* OR interprofession* OR interdisciplinar* OR orthogeriatr* OR co-manag* OR (MH "Interprofessional Relations") OR (MH "Patient Care Team") OR (MH "Intersectoral Collaboration")) | 2,126,567 |
|  | 3. | ("RCT" OR "randomized controlled trial" OR "randomized controlled study" OR "randomly assigned" OR (MH "Randomized Controlled Trials")) | 326,745 |
|  | **Total** | 1 AND 2 AND 3 | 131 |
| **CINAHL Ultimate** |  | ("older adults" OR "elderly" OR "geriatric" OR "aging" OR "senior" OR (MH "Aged")) AND ("hip fracture" OR "hip fractures" OR "hip surgery" OR "hip injury" OR (MH "Hip Fractures")) |  |
|  |  | ("integrated care" OR "collaboration" OR "multi-disciplinary" OR "integrated approach" OR "interdisciplinary" OR "comprehensive" OR team* OR cooperat* OR collaborat* OR transprofession* OR transdisciplinar* OR multiprofession* OR multidisciplinar* OR interprofession* OR interdisciplinar* OR orthogeriatr* OR co-manag* OR (MH "Interprofessional Relations") OR (MH "Patient Care Team") OR (MH "Intersectoral Collaboration")) |  |
|  |  | ("RCT" OR "randomized controlled trial" OR "randomized controlled study" OR "randomly assigned" OR (MH "Randomized Controlled Trials")) |  |
|  | **Total** | 1 AND 2 AND 3 | 84 |
| **Web of Science** | 1. | TS=("older adults" OR "elderly" OR "geriatric" OR "aging" OR "senior" OR "aged") AND TS=("hip fracture" OR "hip fractures" OR "hip surgery" OR "hip injury") | 17,431 |
|  | 2. | TS=("integrated care" OR "collaboration" OR "multi-disciplinary" OR "integrated approach" OR "interdisciplinary" OR "comprehensive" OR team* OR cooperat* OR collaborat* OR transprofession* OR transdisciplinar* OR multiprofession* OR multidisciplinar* OR interprofession* OR interdisciplinar* OR orthogeriatr* OR co-manag*) | 2,144,664 |
|  | 3. | TS=("RCT" OR "randomized controlled trial" OR "randomized controlled study" OR "randomly assigned") | 382,643 |
|  | **Total** | 1 AND 2 AND 3 | 197 |
| **Embase** | 1. | 'older adult':ti,ab OR elderly:ti,ab OR geriatric:ti,ab OR aging:ti,ab OR senior:ti,ab OR 'aged'/exp | 4,793,427 |
|  | 2. | 'hip fracture':ti,ab OR 'hip fractures':ti,ab OR 'hip surgery':ti,ab OR 'hip injury':ti,ab OR 'hip fracture'/exp | 73,175 |
|  | 3. | 'integrated care':ti,ab OR collaboration:ti,ab OR 'multi-disciplinary':ti,ab OR 'integrated approach':ti,ab OR interdisciplinary:ti,ab OR comprehensive:ti,ab OR team*:ti,ab OR cooperat*:ti,ab OR collaborat*:ti,ab OR transprofession*:ti,ab OR transdisciplinar*:ti,ab OR multiprofession*:ti,ab OR multidisciplinar*:ti,ab OR interprofession*:ti,ab OR interdisciplinar*:ti,ab OR orthogeriatr*:ti,ab OR 'co-manag*':ti,ab OR 'interprofessional relation' OR 'patient care team'/exp OR 'intersectoral collaboration'/exp | 1,906,917 |
|  | 4. | 'randomized controlled trial':ti,ab OR 'randomized controlled study':ti,ab OR 'randomly assigned':ti,ab OR rct:ti,ab OR 'randomized controlled trial'/exp | 1,236,838 |
|  | **Total** | 1 AND 2 AND 3 AND 4 | 290 |
| **Scopus** | **Total** | TITLE-ABS-KEY ( "older adults" OR elderly OR geriatric OR aging OR senior OR aged ) AND TITLE-ABS-KEY ( "hip fracture" OR "hip fractures" OR "hip surgery" OR "hip injury" ) AND TITLE-ABS-KEY ( "integrated care" OR collaboration OR "multi-disciplinary" OR "integrated approach" OR interdisciplinary OR comprehensive OR team* OR cooperat* OR collaborat* OR transprofession* OR transdisciplinar* OR multiprofession* OR multidisciplinar* OR interprofession* OR interdisciplinar* OR orthogeriatr* OR "co-manag*" ) AND TITLE-ABS-KEY ( "randomized controlled trial" OR "randomized controlled study" OR "randomly assigned" OR RCT ) | 324 |
| **Cochrane Library** | **Total** | #1 older adults OR elderly OR geriatric OR aging OR senior OR aged  #2 hip fracture OR hip fractures OR hip surgery OR hip injury  #3 integrated care OR collaboration OR multi-disciplinary OR integrated approach OR interdisciplinary OR comprehensive OR team* OR cooperat* OR collaborat* OR transprofession* OR transdisciplinar* OR multiprofession* OR multidisciplinar* OR interprofession* OR interdisciplinar* OR orthogeriatr* OR co NEXT manag*  #4 randomized controlled trial OR randomized controlled study OR randomly assigned OR RCT  #5 MeSH descriptor: [Aged] explode all trees  #6 MeSH descriptor: [Hip Fractures] explode all trees  #7 MeSH descriptor: [Patient Care Team] explode all trees  #8 MeSH descriptor: [Interprofessional Relations] explode all trees  #9 MeSH descriptor: [Intersectoral Collaboration] explode all trees  #10 MeSH descriptor: [Randomized Controlled Trials] explode all trees  #11 #1 OR #5  #12 #2 OR #6  #13 #3 OR #7 OR #8 OR #9  #14 #4 OR #10  #15 #11 AND #12 AND #13 AND #14 | 349 |

***Note***. Last Search November 21, 2025

# **Supplementary Table 5.** Reasons Studies were Excluded During Full-Text Screening (n= 21)

| **No.** | **Article** | **Exclusion reason** |
| --- | --- | --- |
| 1 | Dorotka, R., Schoechtner, H., & Buchinger, W. (2003). Auswirkungen von in der Nacht durchgeführten Stabilisierungsoperationen bei hüftnahen Femurfrakturen auf Mortalitätsrate und Komplikationen [Influence of nocturnal surgery on mortality and complications in patients with hip fractures]. *Der Unfallchirurg*, *106*(4), 287–293. https://doi.org/10.1007/s00113-002-0549-6 | Irrelevant language |
| 2 | Aftab, A., Awan, W. A., Habibullah, S., & Lim, J. Y. (2020). Effects of fragility fracture integrated rehabilitation management on mobility, activity of daily living and cognitive functioning in elderly with hip fracture. *Pakistan journal of medical sciences*, *36*(5), 965–970. https://doi.org/10.12669/pjms.36.5.2412 | Mixed population |
| 3 | Baker, P., Coole, C., Drummond, A., Khan, S., McDaid, C., Hewitt, C., Kottam, L., Ronaldson, S., Coleman, E., McDonald, D. A., Nouri, F., Narayanasamy, M., McNamara, I., Fitch, J., Thomson, L., Richardson, G., & Rangan, A. (2020). Occupational advice to help people return to work following lower limb arthroplasty: the OPAL intervention mapping study. *Health technology assessment (Winchester, England)*, *24*(45), 1–408. https://doi.org/10.3310/hta24450 | Mixed population |
| 4 | Crowe, J., & Henderson, J. (2003). Pre-arthroplasty rehabilitation is effective in reducing hospital stay. *Canadian journal of occupational therapy. Revue canadienne d'ergotherapie*, *70*(2), 88–96. https://doi.org/10.1177/000841740307000204 | Mixed population |
| 5 | Eichler, S., Salzwedel, A., Rabe, S., Mueller, S., Mayer, F., Wochatz, M., Hadzic, M., John, M., Wegscheider, K., & Völler, H. (2019). The Effectiveness of Telerehabilitation as a Supplement to Rehabilitation in Patients After Total Knee or Hip Replacement: Randomized Controlled Trial. *JMIR rehabilitation and assistive technologies*, *6*(2), e14236. https://doi.org/10.2196/14236 | Mixed population |
| 6 | Elley, C. R., Robertson, M. C., Garrett, S., Kerse, N. M., McKinlay, E., Lawton, B., Moriarty, H., Moyes, S. A., & Campbell, A. J. (2008). Effectiveness of a falls-and-fracture nurse coordinator to reduce falls: a randomized, controlled trial of at-risk older adults. *Journal of the American Geriatrics Society*, *56*(8), 1383–1389. https://doi.org/10.1111/j.1532-5415.2008.01802.x | Mixed population |
| 7 | Bell, J. J., Bauer, J. D., Capra, S., & Pulle, R. C. (2014). Multidisciplinary, multi-modal nutritional care in acute hip fracture inpatients - results of a pragmatic intervention. *Clinical nutrition (Edinburgh, Scotland)*, *33*(6), 1101–1107. https://doi.org/10.1016/j.clnu.2013.12.003 | Not interprofessional practice |
| 8 | Borges, F. K., Bhandari, M., Guerra-Farfan, E., Patel, A., Sigamani, A., Umer, M., . . . Wright, J. (2020). Accelerated surgery versus standard care in hip fracture (HIP ATTACK): an international, randomised, controlled trial. *The Lancet, 395*(10225). doi:10.1016/S0140-6736(20)3005 | Not interprofessional practice |
| 9 | Corbella-Giménez, C., Monge-Cid, E., Gallo-Carrasco, A., Barros García-Imhof, J., Sánchez-Rodríguez, F., Díaz-García, J., Vasserot, I., Anadon-Baselga, M. J., & Zaballos, M. (2025). Effect of an Optimized Clinical Pathway Protocol Including Fascia Iliaca Compartment Block on Delirium and Postoperative Complications in Elderly Hip Fracture Patients. *Journal of Clinical Medicine*, *14*(15), 5284. https://doi.org/10.3390/jcm14155284 | Not interprofessional practice |
| 10 | Fang, K., Sun, L., Ma, Y., Gang, S., Li, L., Zhou, X., & Li, J. (2025). An optimized non-opioid multimodal analgesia regimen to promote rapid recovery of patients undergoing primary total hip arthroplasty: a randomized controlled trial. *Clinical Nutrition ESPEN*, *65*, 495-496. https://doi.org/10.1016/j.clnesp.2024.10.018 | Not interprofessional practice |
| 11 | Rittharomya, J., Krua-aum, P., & Aree-Ue, S. (2024). Effectiveness of a Rehabilitation Self-Efficacy Program on Postoperative Outcomes in Older Adults with Hip Fracture: A Randomized Controlled Trial. Pacific Rim International Journal of Nursing Research, 29(1), 91–107. https://doi.org/10.60099/prijnr.2025.269063 | Not interprofessional practice |
| 12 | Breedveld-Peters, J. J., Reijven, P. L., Wyers, C. E., van Helden, S., Arts, J. J., Meesters, B., Prins, M. H., van der Weijden, T., & Dagnelie, P. C. (2012). Integrated nutritional intervention in the elderly after hip fracture. A process evaluation. *Clinical nutrition (Edinburgh, Scotland)*, *31*(2), 199–205. https://doi.org/10.1016/j.clnu.2011.10.004 | Irrelevant design (process evaluation) |
| 13 | Backhouse, T., Fox, C., Hammond, S. P., Poland, F., McDermott-Thompson, V., Penhale, B., & Cross, J. L. (2023). Implementing an intervention to enhance care delivery and consistency for people with hip fracture and cognitive impairment in acute hospital wards: a mixed methods process evaluation of a randomised controlled feasibility trial (PERFECTED). *BMJ open*, *13*(2), e064482. https://doi.org/10.1136/bmjopen-2022-064482 | Irrelevant design (process evaluation) |
| 14 | Chuan, A., Zhao, L., Tillekeratne, N., Alani, S., Middleton, P. M., Harris, I. A., McEvoy, L., & Ní Chróinín, D. (2020). The effect of a multidisciplinary care bundle on the incidence of delirium after hip fracture surgery: a quality improvement study. *Anaesthesia*, *75*(1), 63–71. https://doi.org/10.1111/anae.14840 | Irrelevant design (Quality-improvement study) |
| 15 | Cross, J. L., Hammond, S. P., Shepstone, L., Poland, F., Henderson, C., Backhouse, T., Penhale, B., Donell, S., Knapp, M., Lewins, D., MacLullich, A., Patel, M., Sahota, O., Smith, T. O., Waring, J., Howard, R., Ballard, C., & Fox, C. (2022). PERFECTED enhanced recovery pathway (PERFECT-ER) versus standard acute hospital care for people after hip fracture surgery who have cognitive impairment: a feasibility cluster randomised controlled trial. *BMJ open*, *12*(2), e055267. https://doi.org/10.1136/bmjopen-2021-055267 | Irrelevant design (Feasibility trial) |
| 16 | Flikweert, E. R., Izaks, G. J., Reininga, I. H., Wendt, K. W., & Stevens, M. (2013). Evaluation of the effect of a comprehensive multidisciplinary care pathway for hip fractures: design of a controlled study. *BMC musculoskeletal disorders*, *14*(1), 291. https://doi.org/10.1186/1471-2474-14-291 | Irrelevant design (Study protocol (non-randomized design)) |
| 17 | Flikweert, E. R., Wendt, K. W., Diercks, R. L., Izaks, G. J., Stewart, R., Stevens, M., & Reininga, I. H. F. (2021). A comprehensive multidisciplinary care pathway for hip fractures better outcome than usual care?. *Injury*, *52*(7), 1819–1825. https://doi.org/10.1016/j.injury.2021.04.044 | Irrelevant design (Non-randomized controlled trial) |
| 18 | Lee, H., & Lee, S. H. (2022). Effectiveness of Multicomponent Home-Based Rehabilitation in Elderly Patients after Hip Fracture Surgery: A Randomized Controlled Trial. *Journal of personalized medicine*, *12*(4), 649. https://doi.org/10.3390/jpm12040649 | Not interprofessional practice |
| 19 | Zhang, J., Yang, M., Zhang, X., He, J., Wen, L., Wang, X., Shi, Z., Hu, S., Sun, F., Gong, Z., Sun, M., Li, Q., Peng, K., Ye, P., Ma, R., Zhu, S., Wu, X., Webster, R. J., Ivers, R. Q., & Tian, M. (2021). The effectiveness of a co-management care model on older hip fracture patients in China - A multicentre non-randomised controlled study. *The Lancet regional health. Western Pacific*, *19*, 100348. https://doi.org/10.1016/j.lanwpc.2021.100348 | Irrelevant design (Non-randomized controlled trial) |
| 20 | Shyu, Y. I., Liang, J., Wu, C. C., Su, J. Y., Cheng, H. S., Chou, S. W., & Yang, C. T. (2005). A pilot investigation of the short-term effects of an interdisciplinary intervention program on elderly patients with hip fracture in Taiwan. Journal of the American Geriatrics Society, 53(5), 811–818. https://doi.org/10.1111/j.1532-5415.2005.53253.x | Pilot study (interim report of the trial later reported in full as Shyu et al., 2008; excluded per protocol) |
| 21 | Tseng, M. Y., Liang, J., Shyu, Y. I., Wu, C. C., Cheng, H. S., Chen, C. Y., & Yang, C. T. (2012). Effects of interdisciplinary intervention on disability and depressive symptoms in older Taiwanese patients with hip fracture: A prospective cohort study. International Journal of Nursing Studies, 49(6), 717–726. | Secondary analysis of the same trial as Shyu et al. (2008); trajectory analysis with no extractable group means/SDs for meta-analysis |

# **Supplementary Table 6.** Characteristics of Included Trials

| **Author / Country** | **Sample Size** | **Mean Age**  **(Years)** | **Frequency, Duration of Intervention, and Program Duration** | **Outcomes measurement and Follow-up Length** | **Key Results** | **Overall**  **Risk of Bias** |
| --- | --- | --- | --- | --- | --- | --- |
| Berggren et al., 2019 / Sweden | Experimental group: 107  Control group: 98 | 82.9 | - Regular rehabilitation, care, and home visits. - 2.5 months | - Length of stay/ In-hospital | Length of stay shorter for intervention (p < .003) | Low |
| Chong et al., 2013 / Singapore | Experimental group: 92  Control group: 70 | 77.92 | - 2.5 hours/day - 0.5 months | - Length of stay/ In-hospital - Activities of daily living: Barthel Index/ 12 months - Quality of life: 12 items of Short Form Health Survey (SF-12)/ 12 months | Reduced median length of stay (p = .009) | Some concerns |
| Crotty et al., 2019 / Australia | Experimental group: 121  Control group: 119 | 88.6 | - 13h over 4 weeks - One meeting/2 weeks - 1 month | - Activities of daily living: Barthel Index/ 12 months - Quality of life: EuroQoL 5-Dimensions and 5-Levels (EQ-5D-5L)/ 1 month | No significant difference in quality of life and activities of daily living | Low |
| Karlsson et al., 2016 / Sweden | Experimental group: 107  Control group: 98 | 82.9 | - Regular rehabilitation, care, and home visits - 2.5 months | - Activities of daily living: Barthel Index/ 12 months | No significant difference in activities of daily living | Low |
| Naglie et al., 2002 / Canada | Experimental group: 141  Control group: 138 | 84.20 | - Physiotherapy twice/day - Interdisciplinary care twice/week - 6 months | - Length of stay/ In-hospital - Activities of daily living: Barthel Index/ 6 months - Mortality/ 6 months | Significant difference in length of stay (p < .001); no difference in activities of daily living or mortality | Low |
| Panella et al., 2018 / Belgium | Experimental group: 301  Control group: 203 | 81.30 | - Not mentioned - 3 months | - Activities of daily living: Barthel Index/ 6 months - Quality of life: EuroQoL 5-Dimensions and 5-Levels (EQ-5D-5L)/ 1 month - Mortality/ 6 months | Mortality statistically significant difference (p < .001); no difference in quality of life or length of stay | Some concerns |
| Prestmo et al., 2015 / Norway | Experimental group: 198  Control group: 199 | 83.30 | - Not mentioned - Not mentioned | - Activities of daily living: Barthel Index/ 12 months - Quality of life: EuroQoL 5-Dimensions and 5-Levels (EQ-5D-5L)/ 4 months | Significant differences in activities of daily living and quality of life at 4 and 12 months (p < .05) | Some concerns |
| Shyu et al., 2016 / Taiwan | Experimental group: 101  Control group: 99 | 76.51 | - Interdisciplinary care: weekly first month, biweekly months 2–4 - Comprehensive geriatric care: monthly first 6 months, bimonthly months 6–12 - Interdisciplinary care: 4 months - Comprehensive geriatric care: 12 months | - Length of stay/ In-hospital - Activities of daily living: Barthel Index/ 24 months - Quality of life: 36 items of Short Form Health Survey (SF-36)/ 24 months | Significant difference in activities of daily living at 6, 12 months (p < .05) and quality of life (p < .01) | Some concerns |
| Shyu et al., 2008 / Taiwan | Experimental group: 80  Control group: 82 | 77.6 | - 30 min/week first month - 30 min/2 weeks months 2–3 - 3 months | - Activities of daily living: Barthel Index/ 3 and 12 months - Mortality/ 3 and 12 months | Improved activities of daily living overtime (p < .05); no difference in mortality | Some concerns |
| Swanson et al., 1998 / Australia | Experimental group: 38  Control group: 33 | 78.17 | - Daily assessment/rehabilitation - Weekly conference - 6 months | - Activities of daily living: Barthel Index/ 6 months - Length of stay/ In-hospital - Mortality/ In-hospital | Significant difference in length of stay (p < .01); no difference in activity of daily living and mortality | Some concerns |
| Vidán et al., 2005 / Spain | Experimental group: 155  Control group: 164 | 81.9 | - Geriatrician visited daily - Rehabilitation specialist planned therapy schedule - 3 months | - Activities of daily living: Barthel Index/ 12 months - Length of stay/ In-hospital - Mortality/ In-hospital | Mortality difference (p < .05); no difference in activities of daily living, length of stay | Low |
| Wu et al., 2023 / China | Experimental group: 38  Control group: 38 | 84.36 | - Preoperative assessment at admission, daily in-hospital care, individualized rehab, remote video follow-up for 12 weeks - 3 months | - Activities of daily living: Harris Hip Score/ 12 months - Length of stay/ In-hospital - Mortality/ 12 months | Higher activities of daily living scores at 1 and 12 months (p < .001); mortality lower but not significant; length of stay shorter (p < .001) | High |

# **Supplementary Table 7.** Interprofessional Collaborative Practice Focus Areas Across Studies

| **Author / Country** | **Professionals Involved** | **Comprehensive Geriatric Assessment** | **Early Surgery** | **Early Mobilization** | **Physiotherapy & Rehabilitation** | **Nutritional Care** | **Social Work** | **Psychological Support** | **Discharge Planning** | **Home / Community Rehabilitation** | **Number of Focal Areas** |
| --- | --- | --- | --- | --- | --- | --- | --- | --- | --- | --- | --- |
| Berggren et al., 2019 / Sweden | Geriatrician, Physiotherapist, Occupational Therapist, Nurse | ✓ |  | ✓ | ✓ |  |  |  | ✓ | ✓ | 5 |
| Chong et al., 2013 / Singapore | Physician, Physiotherapist, Nurse | ✓ | ✓ | ✓ | ✓ |  |  |  |  |  | 4 |
| Crotty et al., 2019 / Australia | Geriatrician, Physiotherapist, Dietitian, Nurse | ✓ |  |  | ✓ | ✓ |  |  |  |  | 3 |
| Karlsson et al., 2016 / Sweden | Geriatrician, Physiotherapist, Occupational Therapist, Nurse | ✓ |  | ✓ | ✓ |  |  |  | ✓ | ✓ | 4 |
| Naglie et al., 2002 / Canada | Geriatrician, Physiotherapist, Nurse | ✓ |  |  | ✓ |  |  |  | ✓ |  | 2 |
| Panella et al., 2018 / Belgium | Surgeon, Anaesthesiologist, Physiotherapist, Nurse | ✓ | ✓ |  | ✓ |  |  |  | ✓ |  | 4 |
| Prestmo et al., 2015 / Norway | Geriatrician, Orthopedic Surgeon, Nurse, Physiotherapist, Occupational Therapist | ✓ | ✓ |  | ✓ |  |  |  | ✓ |  | 4 |
| Shyu et al., 2016 / Taiwan | Geriatrician, Physiotherapist, Nurse | ✓ |  |  | ✓ |  |  |  | ✓ | ✓ | 4 |
| Shyu et al., 2008 / Taiwan | Geriatrician, Physiotherapist, Nurse | ✓ |  |  | ✓ |  |  |  | ✓ | ✓ | 4 |
| Swanson et al., 1998 / Australia | Geriatrician, Physiotherapist, Social Worker, Nurse | ✓ | ✓ | ✓ | ✓ |  | ✓ |  | ✓ |  | 6 |
| Vidán et al., 2005 / Spain | Geriatrician, Physiotherapist, Social Worker, Nurse | ✓ |  |  | ✓ |  | ✓ |  | ✓ |  | 4 |
| Wu et al., 2023 / China | Geriatrician, Orthopedic Surgeon, Cardiologist, Respiratory Physician, Anaesthesiologist, Nurse, Neurologist, Physiotherapist, Nutritionist, Psychologist | ✓ | ✓ | ✓ | ✓ |  |  | ✓ | ✓ | ✓ | 7 |
| **Frequency (n)** | — | **12** | **5** | **4** | **12** | **1** | **2** | **1** | **11** | **5** |  |

# **Supplementary Table 8.** Use of the Hip Fracture Core Outcome Set

| **Author / Country** | **Mortality** | **Pain** | **Activity of daily living** | **Mobility** | **Quality of life** | **Number core outcomes set** |
| --- | --- | --- | --- | --- | --- | --- |
| Berggren et al., 2019 / Sweden |  |  |  |  |  | **0** |
| Chong et al., 2013 / Singapore |  |  | ✓ |  | ✓ | **2** |
| Crotty et al., 2019 / Australia | ✓ | ✓ | ✓ |  | ✓ | **4** |
| Karlsson et al., 2016 / Sweden |  |  |  | ✓ |  | **1** |
| Naglie et al., 2002 / Canada | ✓ |  | ✓ |  |  | **2** |
| Panella et al., 2018 / Belgium | ✓ |  | ✓ | ✓ | ✓ | **4** |
| Prestmo et al., 2015 / Norway |  |  | ✓ | ✓ | ✓ | **3** |
| Shyu et al., 2016 / Taiwan |  | ✓ | ✓ | ✓ | ✓ | **4** |
| Shyu et al., 2008 / Taiwan | ✓ |  | ✓ |  |  | **2** |
| Swanson et al., 1998 / Australia | ✓ |  | ✓ |  |  | **2** |
| Vidán et al., 2005 / Spain | ✓ |  | ✓ |  |  | **2** |
| Wu et al., 2023 / China | ✓ |  | ✓ |  |  | **2** |
| **Total** | **7** | **2** | **10** | **4** | **5** |  |

**Supplementary Table 9.** Conceptual and Operational Definitions of All Moderators Used in Subgroup and Meta-Regression Analyses

| **Moderator** | **Type** | **Analysis approach** | **Conceptual definition** | **Operational definition** | **Outcomes examined** |
| --- | --- | --- | --- | --- | --- |
| **Care setting** | Categorical (binary) | Subgroup (descriptive) | The organisational locus and phase span of ICP delivery, distinguishing trials confined to the inpatient admission from those that explicitly extended collaborative practice across the hospital–community boundary. | Inpatient only: all ICP components delivered within the acute or subacute hospital admission; no protocol-defined post-discharge collaborative component. Inpatient + post-discharge: intervention included ≥1 structured post-discharge element (home visits, outpatient interdisciplinary reviews, remote follow-up) delivered by the ICP team. Source: setting and care phases columns of Table S4c. | ADL ≤6m, ADL 12m, LOS, 6m Mortality |
| **Study era** | Categorical (binary) | Subgroup (descriptive/formal for LOS) | The temporal period of trial conduct, reflecting secular changes in orthogeriatric care models, clinical guidelines, and health-system context that may moderate ICP effectiveness. | Dichotomised at 2010 based on publication of landmark orthogeriatric guidelines (British Geriatrics Society, 2007; NICE, 2011) and the Blue Book standards. Pre-2010: primary paper published or recruitment midpoint before 2010. 2010+: otherwise. Shyu 2008 classified as Pre-2010 (recruitment midpoint 2003). For LOS, k≥2 in both cells (formal); for ADL pools, k=1 in Pre-2010 cell (descriptive only). | ADL ≤6m, ADL 12m, LOS, 6m Mortality |
| **Geographic region** | Categorical (multi-level) | Subgroup (formal for LOS; descriptive for ADL) | World region of data collection, reflecting differences in healthcare system structure, staffing norms, hospital LOS culture, and rehabilitation infrastructure that may moderate the size or direction of ICP effects. | East Asia = China (Wu 2023), Singapore (Chong 2013), Taiwan (Shyu 2008, Shyu 2016). Europe = Belgium/Italy/Portugal (Panella 2018), Norway (Prestmo 2015), Spain (Vidán 2005), Sweden (Berggren 2019). Oceania = Australia (Crotty 2019, Swanson 1998). North America = Canada (Naglie 2002). LOS analysis restricted to East Asia vs Europe (k=3 each); other regions are single-study and descriptive only. | ADL ≤6m, ADL 12m, LOS |
| **Geriatrician involvement** | Categorical (binary) | Subgroup (descriptive) | Whether a geriatric medicine specialist was a named, active member of the core ICP team, reflecting the hypothesis that geriatrician co-management amplifies functional and organisational benefits. | Yes: geriatrician or geriatric medicine specialist explicitly named in the methods as part of the core ICP team with responsibility for patient assessment and/or care plan co-development. No/unclear: intervention coordinated via clinical pathway or non-geriatrician coordinator without explicit geriatrician co-management (Chong 2013, Panella 2018). Source: Table S4b team composition fields. | ADL ≤6m, ADL 12m, QoL |
| **Collaboration intensity (binary)** | Categorical (binary) | Subgroup (formal for LOS) | The breadth of interprofessional collaborative practice operationalised in the trial, reflecting the hypothesis that interventions engaging more ICP domains produce larger clinical and health-system effects. | Based on ICP focal areas count from Table S4b (range 2–7). High intensity: ≥5 focal areas (Berggren 2019=5, Swanson 1998=6, Wu 2023=7). Standard: ≤4 focal areas (all others). Note: all ADL and QoL pool studies scored ≤4; evaluable for LOS only. The five ICP focal areas are: M1 shared care plan, M2 joint rounds, M3 integrated documentation, M4 designated coordinator, plus additional trial-specific mechanisms per WHO (2010). | LOS only (no variation in ADL, QoL) |
| **ICP focal areas (continuous count)** | Continuous (integer, 2–7) | Meta-regression (exploratory, LOS) | Number of distinct ICP organisational domains operationalised in the trial, used as a continuous dose-indicator to test whether a linear relationship exists between collaboration breadth and LOS reduction. | Raw integer count from Table S4b (WHO 2010 framework). Centred at the pool mean (4.67) for interpretability. LOS pool values: Berggren=5, Panella=4, Shyu 2008=4, Shyu 2016=4, Vidán=4, Wu 2023=7. Limitation: 5 of 6 studies score 4–5; Wu 2023 is the sole outlier at 7. Regression should be interpreted with caution given limited spread. | LOS only (k=6; exploratory) |
| **Publication year (continuous)** | Continuous (calendar year) | Meta-regression (exploratory, LOS) | Calendar year of primary publication, as a continuous proxy for secular trends in orthogeriatric care intensity, co-intervention contamination, and health-system context that may explain temporal heterogeneity. | Year of publication of the primary outcome paper contributing LOS data. Centred at pool mean (2014.8). LOS pool values: Vidán 2005=2005, Shyu 2008=2008, Shyu 2016=2016, Panella 2018=2018, Berggren 2019=2019, Wu 2023=2023. Directly addresses Reviewer 2 concern about temporal attenuation of ICP effects. | LOS only (k=6; exploratory) |
| **Mean patient age (continuous, years)** | Continuous (years) | Meta-regression (exploratory, LOS) | The mean age of the trial sample, reflecting the hypothesis that older (frailer) patients derive greater benefit from coordinated ICP due to higher baseline complexity and greater susceptibility to care fragmentation. | Overall weighted mean age of the combined ICP and control groups, calculated from published group-level means and sample sizes. Centred at pool mean (80.76 years) for interpretability. Corrected values used throughout (Swanson 1998 = 78.17, correcting the 81.17 misprint). LOS pool values: Berggren=82.9, Panella=81.3, Shyu 2008=77.6, Shyu 2016=76.5, Vidán=81.9, Wu=84.4. | LOS only (k=6; exploratory) |
| **Risk of bias** | Categorical (binary) | Subgroup (formal LOS; descriptive ADL/Mort) | Methodological quality of the trial assessed using the Cochrane RoB 2 tool, used as a sensitivity moderator to evaluate whether pooled effect estimates are robust to inclusion of trials with methodological concerns. | Coded as Low risk of bias or Some concerns based on overall RoB 2 domain ratings reported in the manuscript risk-of-bias table. Low: Berggren 2019, Chong 2013, Crotty 2019, Naglie 2002, Panella 2018, Prestmo 2015, Shyu 2016, Vidán 2005. Some concerns: Shyu 2008, Swanson 1998, Wu 2023. Note: no trial was rated as High risk. QoL pool has no variation (all Low). In-hospital mortality pool has k=1 per cell (not evaluable). | LOS (formal), ADL ≤6m, ADL 12m, 6m Mortality (descriptive) |

**Note.** ICP = interprofessional collaborative practice; WHO = World Health Organization; LOS = length of stay; ADL = activities of daily living; QoL = quality of life; RR = risk ratio; MD = mean difference; RoB = risk of bias. All moderators coded independently by the first and second authors; discrepancies resolved by consensus.

**Supplementary Table 10.** Exploratory Subgroup Analyses for All Outcomes: Interprofessional Collaborative Practice Versus Usual Care in Hip Fracture

| **Outcome [approach]** | **Moderator** | **Subgroup** | **k** | **RE estimate** | **95% CI** | ***p*** | **I²** | **Qbetween (p)** |
| --- | --- | --- | --- | --- | --- | --- | --- | --- |
| **LOS (MD, days) [Formal]** | *Study era* | Pre-2010 | 2 | −1.16 | [−2.95, +0.64] | .207 | 60.3% |  |
|  |  | 2010+ | 4 | −2.85 | [−4.71, −0.99] | .003 | 86.0% |  |
|  |  |  |  |  |  |  |  | *Q(1) = 1.65, p = .198* |
|  | *Geographic region* | East Asia | 3 | −1.45 | [−3.69, +0.78] | .203 | 89.6% |  |
|  |  | Europe | 3 | −3.14 | [−5.09, −1.18] | .002 | 72.9% |  |
|  |  |  |  |  |  |  |  | *Q(1) = 1.24, p = .266* |
|  | *Collaboration intensity* | High (≥5 focal areas) | 2 | −4.42 | [−6.86, −1.98] | < .001 | 69.7% |  |
|  |  | Standard (≤4 focal areas) | 4 | −1.26 | [−2.27, −0.26] | .014 | 48.3% |  |
|  |  |  |  |  |  |  |  | *Q(1) = 5.50, p = .019** |
|  | *Risk of bias* | Low risk | 4 | −2.42 | [−4.17, −0.67] | .007 | 79.8% |  |
|  |  | Some concerns | 2 | −1.89 | [−5.10, +1.32] | .249 | 90.4% |  |
|  |  |  |  |  |  |  |  | *Q(1) = 0.08, p = .775* |
| **ADL ≤ 6 Months (MD) [Descriptive]** | *Care setting* | †Inpatient only | 1 | +2.60 | [−3.76, +8.96] | .423 | — |  |
|  |  | Inpatient + post-discharge | 3 | +4.92 | [+3.08, +6.77] | < .001 | 29.0% |  |
|  |  |  |  |  |  |  |  | *Q(1) = 0.47, p = .492* |
|  | *Study era* | †Pre-2010 | 1 | +7.76 | [+3.00, +12.52] | .001 | — |  |
|  |  | 2010+ | 3 | +4.93 | [+4.62, +5.24] | < .001 | 0.0% |  |
|  |  |  |  |  |  |  |  | *Q(1) = 1.35, p = .245* |
|  | *Geographic region* | East Asia | 3 | +4.30 | [+0.55, +8.06] | .025 | 37.2% |  |
|  |  | †Europe | 1 | +4.95 | [+4.64, +5.26] | < .001 | — |  |
|  |  |  |  |  |  |  |  | *Q(1) = 0.11, p = .736* |
|  | *Geriatrician involvement* | †No geriatrician | 1 | +2.60 | [−3.76, +8.96] | .423 | — |  |
|  |  | Geriatrician involved | 3 | +4.92 | [+3.08, +6.77] | < .001 | 29.0% |  |
|  |  |  |  |  |  |  |  | *Q(1) = 0.47, p = .492* |
|  | *Risk of bias* | Low risk | 3 | +4.93 | [+4.62, +5.24] | < .001 | 0.0% |  |
|  |  | †Some concerns | 1 | +7.76 | [+3.00, +12.52] | .001 | — |  |
|  |  |  |  |  |  |  |  | *Q(1) = 1.35, p = .245* |
| **ADL 12 Months (MD) [Descriptive]** | *Care setting* | †Inpatient only | 1 | −0.70 | [−7.73, +6.33] | .845 | — |  |
|  |  | Inpatient + post-discharge | 3 | +4.88 | [+2.44, +7.31] | < .001 | 37.0% |  |
|  |  |  |  |  |  |  |  | *Q(1) = 2.16, p = .142* |
|  | *Study era* | †Pre-2010 | 1 | +6.17 | [−0.41, +12.75] | .066 | — |  |
|  |  | 2010+ | 3 | +3.13 | [−0.89, +7.14] | .127 | 68.0% |  |
|  |  |  |  |  |  |  |  | *Q(1) = 0.60, p = .439* |
|  | *Geographic region* | East Asia | 3 | +2.19 | [−1.30, +5.69] | .219 | 7.2% |  |
|  |  | †Europe | 1 | +5.65 | [+5.32, +5.98] | < .001 | — |  |
|  |  |  |  |  |  |  |  | *Q(1) = 3.73, p = .053* |
|  | *Geriatrician involvement* | †No geriatrician | 1 | −0.70 | [−7.73, +6.33] | .845 | — |  |
|  |  | Geriatrician involved | 3 | +4.88 | [+2.44, +7.31] | < .001 | 37.0% |  |
|  |  |  |  |  |  |  |  | *Q(1) = 2.16, p = .142* |
|  | *Risk of bias* | Low risk | 3 | +3.13 | [−0.89, +7.14] | .127 | 68.0% |  |
|  |  | †Some concerns | 1 | +6.17 | [−0.41, +12.75] | .066 | — |  |
|  |  |  |  |  |  |  |  | *Q(1) = 0.60, p = .439* |
| **QoL (Hedges' G) [Descriptive]** | *Geriatrician involvement* | †No geriatrician | 1 | +0.051 | [−0.152, +0.254] | .625 | — |  |
|  |  | Geriatrician involved | 2 | +0.279 | [+0.122, +0.436] | < .001 | 0.0% |  |
|  |  |  |  |  |  |  |  | *Q(1) = 3.03, p = .082* |
| **6-Month Mortality (RR) [Descriptive]** | *Study era* | Pre-2010 | 2 | 0.732 | [0.413, 1.299] | .287 | 0.0% |  |
|  |  | †2010+ | 1 | 1.742 | [0.845, 3.592] | .133 | — |  |
|  |  |  |  |  |  |  |  | *Q(1) = 3.39, p = .066* |
|  | *Care setting* | †Inpatient only | 1 | 0.792 | [0.437, 1.436] | .443 | — |  |
|  |  | Inpatient + post-discharge | 2 | 0.500 | [0.050, 5.041] | .556 | 62.9% |  |
|  |  |  |  |  |  |  |  | *Q(1) = 0.01, p = .906* |
|  | *Risk of bias* | Low risk | 2 | 1.142 | [0.529, 2.468] | .735 | 63.2% |  |
|  |  | †Some concerns | 1 | 0.256 | [0.029, 2.243] | .219 | — |  |
|  |  |  |  |  |  |  |  | *Q(1) = 1.62, p = .203* |

**Note.** Subgroup analyses are exploratory and were conducted post hoc in response to peer review. RE = DerSimonian–Laird random-effects model. MD = mean difference (positive values favour ICP). RR = risk ratio (values < 1.0 favour ICP). G = Hedges' corrected standardised mean difference. I² = percentage of variance attributable to between-study heterogeneity.

**Q** between = chi-square test for between-subgroup differences (df in parentheses). Formal analyses (LOS) had k ≥ 2 in all cells. Descriptive analyses had k = 1 in ≥1 cell and should not be interpreted as formal statistical comparisons.

**†** Single-study subgroup; estimate is the unadjusted study-level value. Between-subgroup Q is unreliable when any cell contains k = 1.

***** Statistically significant between-subgroup difference: LOS × collaboration intensity, Q(1) = 5.50, p = .019. High-intensity trials (Berggren 2019; Wu 2023) showed greater LOS reduction (MD = −4.42 days) than standard-intensity trials (MD = −1.26 days).

**Supplementary Table 11.** Exploratory Meta-Regression Analyses for Length of Stay (k = 6): ICP Versus Usual Care in Hip Fracture

| **Predictor (LOS, k = 6)** | **β₀** | **β₁ (slope)** | **95% CI for β₁** | ***t*(4)** | ***p*** | **τ² (null)** | **τ² (residual)** | **R²ₐ** | **Q_res (p)** |
| --- | --- | --- | --- | --- | --- | --- | --- | --- | --- |
| Publication year (centred at 2014.8 years) | −2.24 | −0.147 | [−0.438, +0.144] | −1.407 | .232 | 2.330 | 2.077 | 10.9% | 5.03 (.284) |
| ICP focal areas count (centred at 4.67 areas) | −2.19 | −0.817 | [−2.275, +0.642] | −1.555 | .195 | 2.330 | 1.708 | 26.7% | 5.54 (.237) |
| Mean patient age (centred at 80.76 years) | −2.14 | **−0.447** | **[−0.810, −0.083]** | **−3.413** | **.027** | 2.330 | **0.372** | **84.0%** | 4.98 (.290) |

**Note.** All analyses used weighted least squares with DerSimonian–Laird random-effects weights (1/[σ²ᵢ + τ²_null]). Predictors were mean-centred prior to entry. β₀ = intercept (pooled LOS MD at the mean of the predictor). β₁ = slope (change in pooled LOS MD per 1-unit increase in predictor).

**t**(4) and 95% CI for β₁ use the t-distribution with df = k − 2 = 4. τ² (null) = between-study variance from the unconditional model. τ² (residual) = between-study variance after accounting for the predictor. R²ₐ = proportional τ² reduction: max(0, [τ²_null − τ²_res] / τ²_null × 100). Q_res = residual heterogeneity test (χ², df = k − 2). Bold values indicate statistical significance (p < .05).

**Limitations.** k = 6 is below the conventional minimum of k = 10 per predictor; all results are exploratory. For ICP focal areas, 5 of 6 studies scored 4–5 (limited spread); Wu 2023 (7 focal areas) exerts disproportionate leverage on that estimate.
